# Transformative skeletal motion analysis: optimization of exercise training and injury prevention through graph neural networks

**DOI:** 10.3389/fnins.2024.1353257

**Published:** 2024-03-28

**Authors:** Jiaju Zhu, Zijun Ye, Meixue Ren, Guodong Ma

**Affiliations:** ^1^School of Physical Education, Northeast Normal University, Changchun, Jilin, China; ^2^College of Life and Health Sciences, The Chinese University of Hong Kong, Hong Kong, China; ^3^Graduate School, Jilin Sport University, Changchun, Jilin, China; ^4^Human Movement Science College, Jilin Sport University, Changchun, Jilin, China; ^5^Sports Prescription Department, Dongshin University, Naju, Jeollanam-do, Republic of Korea

**Keywords:** injury prediction, training optimization, assistive devices, Transformer, Generative Adversarial Network

## Abstract

**Introduction:**

Exercise is pivotal for maintaining physical health in contemporary society. However, improper postures and movements during exercise can result in sports injuries, underscoring the significance of skeletal motion analysis. This research aims to leverage advanced technologies such as Transformer, Graph Neural Networks (GNNs), and Generative Adversarial Networks (GANs) to optimize sports training and mitigate the risk of injuries.

**Methods:**

The study begins by employing a Transformer network to model skeletal motion sequences, facilitating the capture of global correlation information. Subsequently, a Graph Neural Network is utilized to delve into local motion features, enabling a deeper understanding of joint relationships. To enhance the model's robustness and adaptability, a Generative Adversarial Network is introduced, utilizing adversarial training to generate more realistic and diverse motion sequences.

**Results:**

In the experimental phase, skeletal motion datasets from various cohorts, including professional athletes and fitness enthusiasts, are utilized for validation. Comparative analysis against traditional methods demonstrates significant enhancements in specificity, accuracy, recall, and *F*1-score. Notably, specificity increases by ~5%, accuracy reaches around 90%, recall improves to around 91%, and the *F*1-score exceeds 89%.

**Discussion:**

The proposed skeletal motion analysis method, leveraging Transformer and Graph Neural Networks, proves successful in optimizing exercise training and preventing injuries. By effectively amalgamating global and local information and integrating Generative Adversarial Networks, the method excels in capturing motion features and enhancing precision and adaptability. Future research endeavors will focus on further advancing this methodology to provide more robust technological support for healthy exercise practices.

## 1 Introduction

With the vigorous development of artificial intelligence technology, computer vision (Voulodimos et al., 2018), as one of its key branches, is rapidly expanding its application areas and continuously enhancing problem-solving capabilities. In this context, skeletal motion analysis, as an important research direction within computer vision, aims to identify ongoing actions such as jumping, clapping, and making phone calls from continuous human skeletal point data. This technology not only finds widespread applications in intelligent surveillance (Sreenu and Durai, 2019), human-computer interaction (Yun et al., 2021), and virtual reality (Maskeliūnas et al., 2023), but also holds significant importance and challenges in areas such as optimizing sports training and preventing injuries.

Exercise, as a crucial means of maintaining physical health, may lead to sports injuries and even permanent damage due to incorrect postures and movements (Emery and Pasanen, 2019). This raises a key question related to the study of neuromusculoskeletal models: How can skeletal motion analysis be employed to enhance exercise effectiveness, reduce potential harm, and optimize and improve the movements of athletes? In exploring this question, we focus on two main aspects: firstly, determining whether the athlete's movements are correct or if there are any adverse habits or potential risks based on skeletal motion data; secondly, providing targeted advice and feedback based on skeletal motion data to help athletes improve and optimize their movements, thereby enhancing efficiency and safety during exercise.

Research in this field plays a crucial role in promoting the application of neuromusculoskeletal models in motion analysis and training optimization (Peng and Li, 2023). The neuromusculoskeletal model is a biomechanical model used to describe the structure and function of the human movement system. This model includes the nervous system, muscular system, and skeletal system, and describes their interactions.

Here is an explanation of each part of the NMBS:

- Nervous system: the nervous system is responsible for transmitting signals and commands to control body movement and actions. It includes the brain, spinal cord, and peripheral nervous system, which transmit information through electrochemical signals between neurons.
- Muscular system: the muscular system comprises muscle tissues, which are made up of muscle fibers and are connected to bones via tendons. Muscles generate force through contraction and relaxation, driving skeletal movement.
- Skeletal system: The skeletal system consists of bones, including bones, joints, and connective tissues. It provides support and structure to the body and serves as the pivot point for muscle movement.

The interactions between these systems form the neuromusculoskeletal system, which controls and regulates human movement. The goal of the NMBS model is to simulate and predict the dynamic relationships between the nervous, muscular, and skeletal systems during human movement, in order to better understand and optimize applications such as human movement, rehabilitation therapy, and sports training.

By combining computer vision and neuromusculoskeletal model techniques, we aim to have a more comprehensive understanding of muscle activity and skeletal motion during exercise, providing more precise assessments and treatment plans for injury prevention and rehabilitation. Such research holds the promise of advancing the practical application of computer simulation in clinical sports therapy, bringing new possibilities for personalized healthcare and sports training.

At the same time, predecessors have conducted in-depth research by employing refined representations of skeletal data, deep extraction of motion features, and innovative design of action classification models. Starting from key issues such as data noise, annotation dependency, and representational capacity, researchers have employed various methods and approaches, bringing forth a series of remarkable solutions to the field of skeletal motion analysis. In the following, we will delve into the specific methods adopted by predecessors in these aspects and the significant achievements they have made, aiming to provide valuable insights for the further development of this research. Currently, in this field, the research by predecessors can be broadly categorized into the following aspects:

- Representation of skeletal data: skeletal data refers to a topological representation of the joints and bones of the human body, typically including two-dimensional or three-dimensional coordinates and confidence levels. Skeletal data can be obtained from depth sensors (such as Kinect) or pose estimation algorithms (such as OpenPose, HRNet, etc.). Compared to RGB or depth images, skeletal data is more compact, robust, and easier to handle, but it also poses challenges such as limited information, noise interference, and data imbalance. Therefore, effectively representing and preprocessing skeletal data is a fundamental and critical issue in motion analysis. Some common methods for representing skeletal data include spatial-temporal graphs (Wu et al., 2019), skeleton sequences, skeleton images (Yang et al., 2018), etc.
- Extraction of motion features: motion features refer to characteristics that reflect the essential properties of actions and differentiate between different actions, typically encompassing spatial and temporal features. Spatial features involve information such as the relative positions, angles, and distances between skeletal points, while temporal features include the variations, velocities, accelerations, etc., of skeletal points over time. Extracting motion features is a core issue in motion analysis and a key factor affecting action recognition performance. Some common methods for extracting motion features include handcrafted features, Convolutional Neural Networks (CNN) (Li et al., 2021), Recurrent Neural Networks (RNN) (Yu et al., 2019), Graph Neural Networks (GNN), etc.
- Models for action classification: action classification involves categorizing actions into different classes based on the extracted motion features, such as jumping, clapping, making a phone call, etc. Action classification is the ultimate goal of motion analysis and a primary metric for evaluating motion analysis methods. Some common models for action classification include Support Vector Machines (SVM) (Ning E. et al., 2024), Random Forest (RF), Multilayer Perceptron (MLP) (Almeida, 2020), Fully Connected Networks (FCN), Attention Mechanism, etc.

After discussing the significant achievements in the field of skeletal motion analysis by previous researchers, we cannot ignore the fact that this field still faces a series of challenges and issues. Firstly, the issue of noise in skeletal motion data directly affects the accuracy and reliability of the data. To address this problem, advanced signal processing techniques and model designs need to be introduced. Secondly, the dependency on annotations for skeletal motion data becomes prominent in supervised learning, making effective use of unlabeled or sparsely labeled data a pressing challenge. Additionally, the problem of representational capacity in skeletal motion data requires us to contemplate how to better model spatiotemporal local feature points to enhance the model's expressive power. Lastly, multi-person detection issues involve complex scenarios such as computational efficiency and occlusion, necessitating more efficient computation methods and strategies for the fusion of multimodal information.

To address the challenges in the field of skeletal motion analysis, we have drawn upon various research findings concerning gait analysis and artificial limb recognition, all of which offer valuable insights. For instance, Weng et al. (2023) proposed a gait stability assessment method based on wearable accelerometer sensors. This method effectively evaluates the balance and stability of gait by analyzing acceleration signals during the gait process, providing strong support for rehabilitation therapy. Additionally, addressing the issue of artificial limb recognition, Li et al. (2020) proposed a method based on surface electromyographic (EMG) signals. By aggregating and processing EMG signals, this method significantly improves the accuracy of artificial limb motion recognition, contributing to the enhancement of mechanical assistive technologies.

In this study, we have employed advanced deep learning techniques, primarily including Transformer, Graph Neural Networks , and Generative Adversarial Networks. The introduction of Transformer networks enables us to comprehensively capture global information in skeletal motions, thereby enhancing our understanding of the overall structure of movement and consequently improving the accuracy of motion quality assessment. Additionally, the integration of Graph Neural Networks helps to model the relationships between skeletal joints more finely, addressing the issue of skeletal data representation capability. Moreover, the application of Generative Adversarial Networks provides effective means for data augmentation and noise reduction, enhancing the model's robustness to noise. The comprehensive application of these methods is expected to significantly enhance the effectiveness of motion training optimization and injury prevention in practice.

This study delves into the critical issues of neuromusculoskeletal models in the field of skeletal motion analysis, not only addressing numerous challenges but also providing innovative methods and strategies for optimizing sports training and preventing injuries. Through efficient motion feature extraction and accurate action classification based on Transformer, Graph Neural Networks, and Generative Adversarial Networks, our research aims to offer athletes more scientific and personalized training guidance, thereby enhancing athletic performance and reducing the incidence of sports-related injuries.

These research outcomes have not only made significant theoretical advancements but also demonstrated outstanding performance in practical applications. By optimizing data representation, extracting motion features, and designing innovative classification models, we anticipate that these research findings will establish a solid foundation for the development of neuromusculoskeletal models. This not only benefits academic research and practical applications in related fields but also opens up new possibilities for the progress of neuromusculoskeletal models in the realms of injury, disease, and clinical treatment. We firmly believe that the outcomes of this study will provide positive insights for making greater breakthroughs in the field of human movement health, paving the way for new directions in both academic research and practical applications in relevant domains.

The contributions of this paper can be summarized in the following three aspects:

1. Pioneered transformer network application in skeletal motion analysis, enhancing global associative information capture for comprehensive motion understanding.
2. Introduced graph neural networks for local motion feature modeling, enabling precise analysis of joint relationships and addressing varied motion scenarios effectively.
3. Integrated generative adversarial networks for realistic and diverse motion sequence generation, enhancing model adaptability and opening new avenues in skeletal motion analysis.

The logical structure of this paper is as follows: In the second section, a literature review was conducted to provide an overview of research and methodologies in the relevant field. The strengths and weaknesses of existing approaches were analyzed, leading to the elucidation of the research motivations and objectives of this paper. The third section, the methodology introduction, meticulously expounds on the three major technical approaches proposed in this study: the first being the Transformer model, the second being the Graph Neural Network model, and the third being the Generative Adversarial Network model. The fourth section, experimental analysis and comparison, provides a detailed description of the experimental datasets, environments, design processes, and the evaluation metric system. By contrasting experimental results, comparing predictive capabilities, training speeds, and model complexities across multiple public datasets, this section elucidates the advantages of the proposed models in this research. In the fifth section, discussion and conclusion, the study's main contributions and areas that need further improvement are systematically summarized. Future research directions are also outlined.

## 2 Related work

Skeletal motion analysis, as an interdisciplinary field, encompasses computer modeling, machine learning, sports biomechanics (Bartlett, 2014), rehabilitation medicine, and various other domains. Its core objective is to enhance exercise effectiveness, reduce sports injuries, and assist in movement rehabilitation by capturing, recognizing, evaluating, and optimizing human skeletal movements. Research in this field provides crucial insights into the advancements of neuromusculoskeletal models in injury, disease, and clinical treatment. With the flourishing development of depth sensors and artificial intelligence technology, significant progress has been made in skeletal motion analysis, closely linked to the application of neuromusculoskeletal models in assessing disease impacts and diagnostics. However, despite these advancements, there are still challenges and issues that not only limit the in-depth development of skeletal motion analysis itself but also affect the application of neuromusculoskeletal models in clinical treatment and rehabilitation. In the following discussion, we will review and analyze work in the field of skeletal motion analysis closely related to our research, elucidating our research motivations and contributions. We will also highlight the potential applications of neuromusculoskeletal models in injury, disease, and clinical treatment. This series of research efforts aims to deepen our understanding of neuromusculoskeletal models and provide new insights for their widespread application in practical medical settings.

In the field of skeletal motion analysis, the traditional approach has been the use of marker systems. However, its limitations include the need for labor-intensive manual labeling, constraints in specific environments, and time-consuming data processing. In this regard, a review article (Colyer et al., 2018) has been proposed, focusing on the evolution of visual motion analysis, particularly emphasizing the transition from traditional marker systems to modern markerless systems. The review highlights the widespread application of current motion analysis systems in sports biomechanics and rehabilitation medicine, but points out their limitations in requiring manual attachment of markers, demanding controlled environments, and involving lengthy data processing. This provides a clear background for our research. Real-time detection, recognition, and assessment of actions are critical issues in skeletal motion analysis. In this context, a paper Patrona et al. (2018) introduces a novel framework aimed at achieving real-time action detection, recognition, and assessment of motion capture data. By utilizing pose and kinematic information, the framework efficiently segments and labels actions. The strength of this paper lies in the adoption of automatic and dynamic weight allocation, changing the importance of joint data based on their involvement in actions, and the use of kinetic-based descriptor sampling. This provides an insight for our research, indicating that better skeletal motion analysis can be achieved through more effective action feature extraction and assessment. Deep sensors play a significant role in skeletal motion analysis, and different versions of sensors exhibit variations in skeletal tracking accuracy and precision. In this domain, a paper Tölgyessy et al. (2021) evaluates the skeletal tracking capabilities of Kinect V1, Kinect V2, and Azure Kinect. Experimental results indicate that Azure Kinect outperforms its predecessors in both accuracy and precision, making it suitable for applications such as human-computer interaction, body motion analysis, and other gesture-based applications. This paper provides crucial information for our hardware selection in skeletal motion analysis. With the availability of large-scale skeletal datasets, 3D human action recognition has become a research hotspot in computer vision. Addressing this issue, a paper Caetano et al. (2019) introduces a novel skeletal image representation called SkeleMotion, used as input for convolutional neural networks. This method enhances the representation of actions by explicitly calculating amplitude and direction values of skeletal joints, aggregating more temporal dynamics across different time scales. It also presents a new direction for exploration in the field of 3D action recognition. In the medical field, predicting and assessing motion injuries are crucial for improving sports safety. In this aspect, a study Song et al. (2021) proposes a deep learning-based Convolutional Neural Network (CNN) method for safety prediction and assessment. Using an optimized CNN model, this method effectively detects and evaluates musculoskeletal disorders, providing robust support for the collection and analysis of medical data. This offers an intriguing perspective for our research, suggesting that combining deep learning and sports medicine can achieve safe prediction and assessment of motion injuries. The application of machine learning methods in motion injury prediction and prevention has become a research focus. In this regard, a review Van Eetvelde et al. (2021) provides a systematic overview of the applications of machine learning in motion injury prediction and prevention. By introducing various machine learning methods, including tree ensemble methods, support vector machines, and artificial neural networks, this review offers in-depth insights into the predictive performance of motion injury. This framework provides an understanding of the potential applications of machine learning in the field of motion injury for our research.

From the above literature review, we can see that skeletal motion analysis is an interdisciplinary field involving multiple domains, with significant theoretical and practical implications. However, existing research methods still have some shortcomings and limitations, primarily manifested in the following aspects:

- Dependency on marker systems: While marker systems can provide high-precision skeletal data, they involve complex operations, requiring specialized equipment and environments, as well as significant human and time costs. Additionally, marker systems are susceptible to factors such as occlusion, noise, and lighting, affecting their robustness and reliability.
- Difficulty in extracting and evaluating motion features: Motion consists of complex spatiotemporal sequences involving multiple joints and limbs, making its features challenging to describe using simple mathematical models or statistical methods. Moreover, motion evaluation needs to consider various factors such as the purpose, type, difficulty, style of the action, as well as individual body conditions, skill levels, psychological states, adding subjectivity, and uncertainty to motion assessment.
- Limitations of machine learning methods: Machine learning methods have extensive applications in predicting and preventing motion injuries but face challenges and issues. For instance, machine learning methods require a substantial amount of annotated data for model training, and obtaining and processing annotated data is a time-consuming and labor-intensive task. Additionally, machine learning methods struggle with handling data imbalances, noise, and anomalies, affecting their generalization and robustness.

In addressing the aforementioned issues, this study proposes a skeleton motion analysis approach based on Transformer and Graph Neural Networks to optimize sports training and enhance injury prevention. Firstly, by introducing the Transformer network, we globally model the skeletal motion sequences, capturing long-term dependencies and contextual information to improve the representation and recognition efficiency of actions. Secondly, with the introduction of Graph Neural Networks, we locally model skeletal motion sequences using a graph structure to describe the topological relationships between joints, enhancing action details and accuracy. Finally, through the use of Generative Adversarial Networks (GANs), we conduct adversarial training on skeletal motion sequences to generate more realistic and diverse action sequences, enhancing the model's robustness and adaptability. Crucially, this study not only focuses on improving sports performance but also emphasizes injury prevention. Leveraging the characteristics of Generative Adversarial Networks, we aim to reduce potential sports injuries while enhancing sports performance, providing comprehensive support for healthy exercise.

Overall, this study has made significant strides in the innovative application of neural-musculoskeletal models. By leveraging both global and local information and introducing Generative Adversarial Networks, we have successfully optimized sports training and prevented sports injuries. The substantial improvements in specificity, accuracy, recall, *F*1-score, and other metrics further confirm the effectiveness of our approach and its unique contributions to the field of neural-musculoskeletal model research. We believe that this new perspective and approach will provide a beneficial supplement to the medical community's focus on injury prevention and sports training in the neural-musculoskeletal system, offering valuable insights for future research directions.

## 3 Methodology

**Figure 1:**
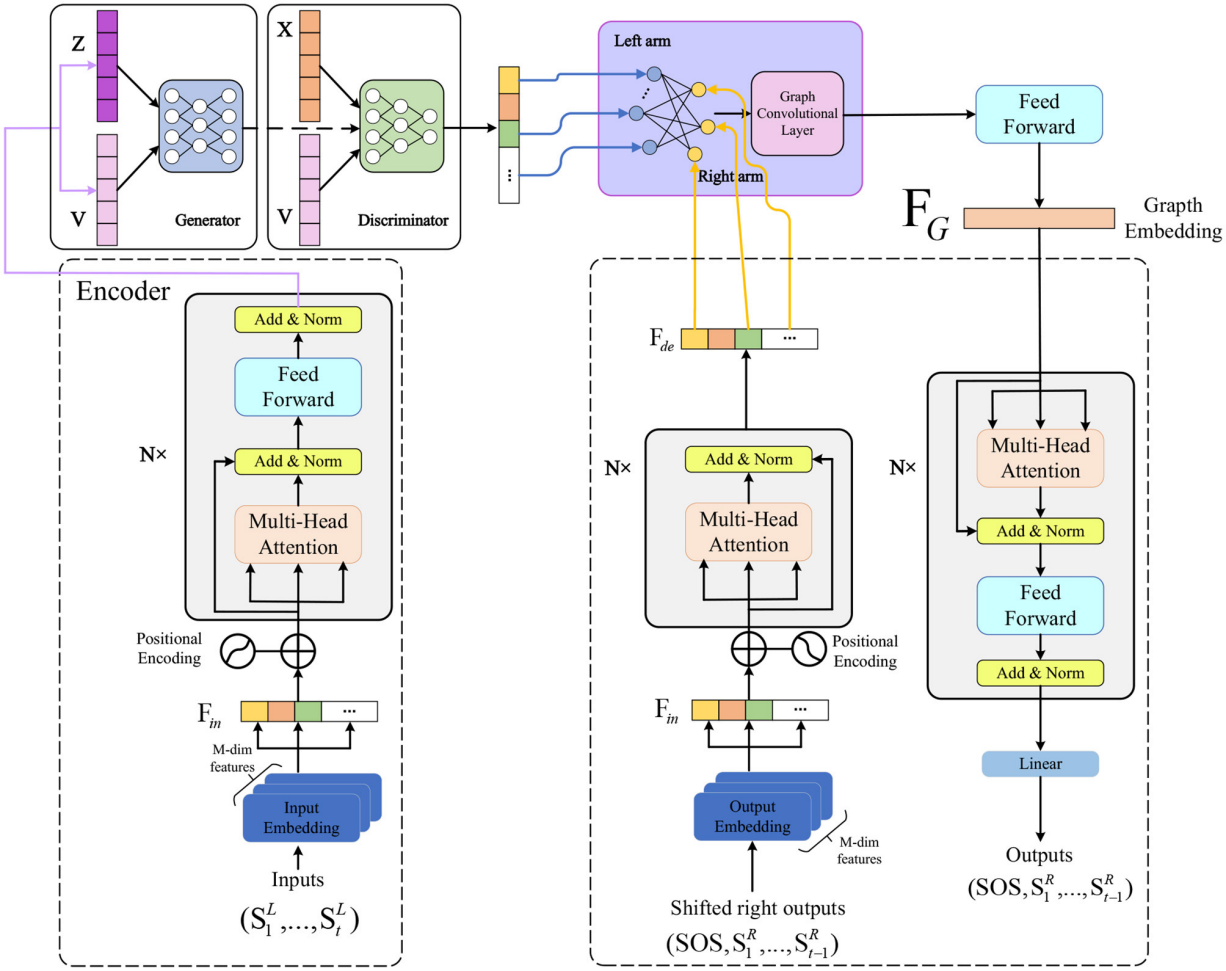
Overall algorithm flowchart.

In the methodology section of this study, we will provide a detailed introduction to the three key methods employed, namely the Transformer model, Graph Neural Networks , and Generative Adversarial Networks . The clever combination of these three methods constitutes our comprehensive skeleton motion analysis framework, aiming to capture global and local correlations comprehensively and enhance the model's adaptability through Generative Adversarial Networks. To illustrate the overall design of our algorithm clearly, we will present the details of each method in the following sections and demonstrate their interaction and integration throughout the entire process using an overall algorithm framework diagram (as shown in Figure 1).

### 3.1 Transformer model

**Figure 2:**
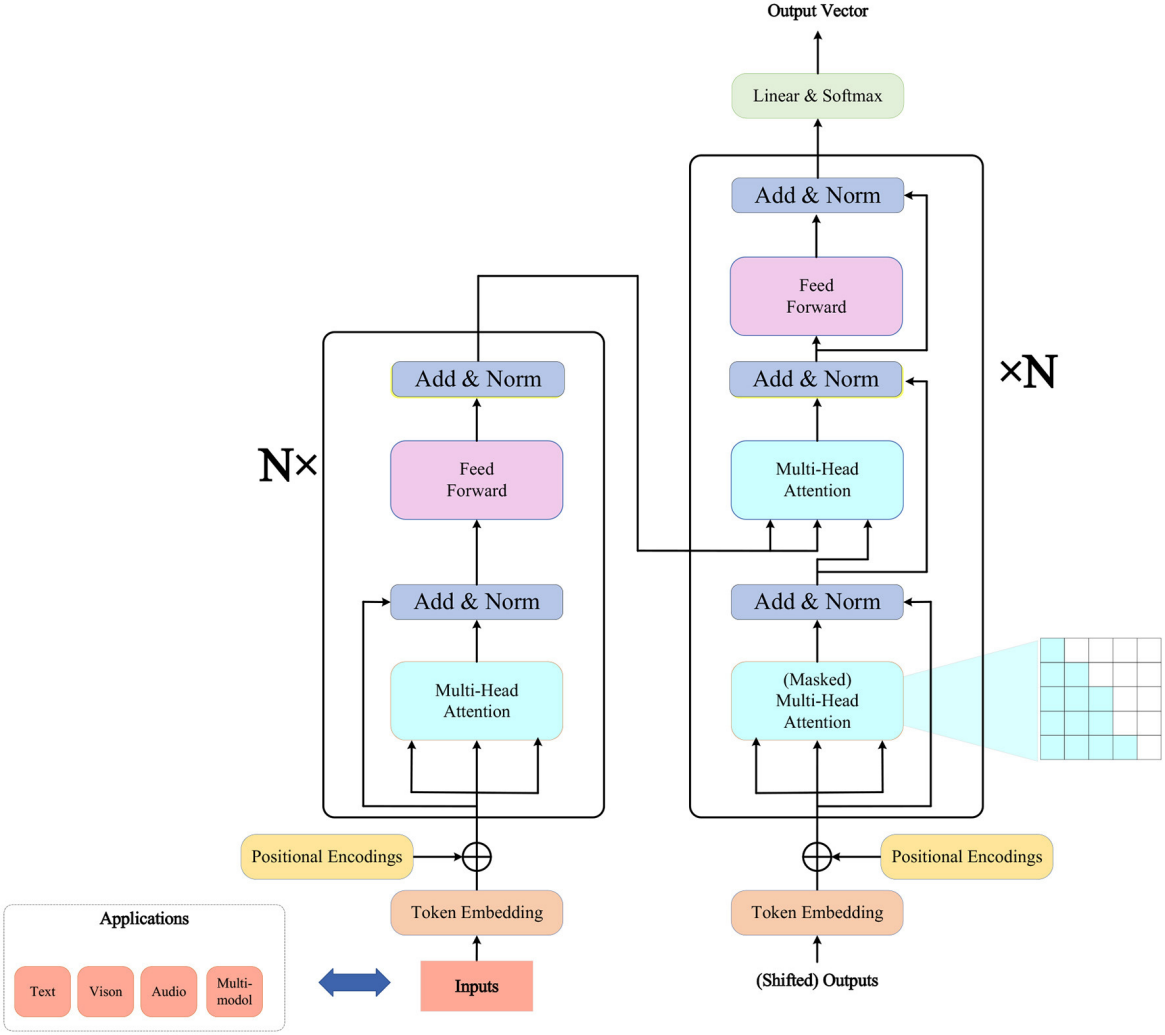
Transformer model.

The Transformer model is a deep learning architecture based on the self-attention mechanism, designed to address sequence-to-sequence tasks in natural language processing, such as machine translation, text summarization, and more (Han et al., 2021). The main characteristic of the Transformer model is the complete departure from traditional Recurrent Neural Networks (RNN) and Convolutional Neural Networks (CNN), opting solely for the self-attention mechanism (Niu et al., 2021) to capture global dependencies within sequences, thereby enhancing model parallelism and efficiency. The overall architecture of the Transformer model is illustrated in Figure 2.

The Transformer model consists of two parts: the encoder and the decoder. The encoder transforms an input sequence (such as a sentence) into a continuous vector representation, while the decoder generates the next output (such as words in another language) based on the encoder's output and the previous output sequence. Both the encoder and decoder are composed of multiple identical layers, each containing two sub-layers: a Multi-Head Attention sub-layer and a Feed-Forward Neural Network sub-layer. Residual connections and layer normalization are applied between the two sub-layers.

The role of the Multi-Head Attention sub-layer is to calculate the correlation between each element (such as a word) in the input sequence and other elements, producing a weighted contextual representation (Tao et al., 2018). This sub-layer comprises several self-attention heads, each performing self-attention calculations on the input sequence. The outputs of all self-attention heads are concatenated and then linearly transformed to obtain the final output. Self-attention is calculated as shown in Equation (1):


(1) $$\begin{array}{l} \text{Attention}\left(Q,K,V\right)=\text{softmax}\left(\frac{QK^{T}}{\sqrt{d_{k}}}\right)V \end{array}$$

Here, *Q*, *K*, and *V* represent the Query, Key, and Value matrices, respectively. They are obtained by subjecting the input sequence to different linear transformations. *d*_*k*_ denotes the dimensionality of the key. The *softmax* function is applied along the last dimension, normalizing each row independently. The meaning of this formula is that, for each query, the dot product (inner product) with all keys is calculated, then scale it by dividing by $\sqrt{d_{k}}$, followed by obtaining a probability distribution using the *softmax* function, representing the attention weights of the query for each key. Finally, this probability distribution is multiplied by the Value matrix to obtain the output for the query. Multi-head self-attention is calculated in Equation (2):


(2) $$\begin{array}{l} \text{MultiHead}\left(Q,K,V\right)=\text{Concat}\left({\text{head}}_{1},\text{...},{\text{head}}_{h}\right)W^{0} \end{array}$$

The matrices $W_{i}^{Q}$, $W_{i}^{K}$, $W_{i}^{V}$, and *W*^*O*^ are all learnable parameter matrices. Concat represents the concatenation operation. The meaning of this formula is that for each self-attention head, a linear transformation is applied to the input sequence using different parameter matrices, followed by self-attention computation to obtain an output. Finally, the outputs of all self-attention heads are concatenated, and a linear transformation is applied to obtain the final output.

The purpose of the feed-forward neural network (FFNN) sublayer is to apply a non-linear transformation to the output of the multi-head self-attention sublayer, enhancing the model's expressive power. The FFNN sublayer consists of two linear transformations and an activation function (such as ReLU), and Equation (3) is given as follows:


(3) $$\begin{array}{l} \text{FFN}\left(x\right)=\max\left(0,x_{1}W_{1}+b_{1}\right)W_{2}+b_{2} \end{array}$$

This formula represents the feed-forward neural network component in the Transformer model. It takes input *x*_1_ and outputs after a series of linear transformations and non-linear activation functions. *W*_1_, *W*_2_, *b*_1_, and *b*_2_ are all learnable parameter matrices or vectors. max(0, •) represents the ReLU activation function.

Each layer of the encoder and decoder has a multi-head self-attention sub-layer and a feed-forward neural network sub-layer. However, the decoder has an additional multi-head self-attention sub-layer called the encoder-decoder attention. This sub-layer computes attention over the encoder's output to integrate information from both the source language and target language. The computation is similar to self-attention, but it uses the encoder's output as keys and values and the decoder's output as queries.

To enable the model to distinguish elements at different positions in the sequence, the Transformer model introduces positional encoding. This involves adding a position-related vector to the vector representation of each element in the input sequence. The position encoding is shown in Equations (4) and (5):


(4) $$\begin{array}{l} \text{PE}\left(pos,2i\right)=\sin\left(\frac{pos}{10000^{2i/d_{model}}}\right) \end{array}$$

(5) $$\begin{array}{l} \text{PE}\left(pos,2i+1\right)=\cos\left(\frac{pos}{10000^{2i/d_{modsl}}}\right) \end{array}$$

In the formula, *pos* represents the position, *i* represents the dimension, and *d*_*model*_ represents the model's dimension. The meaning of this formula is that for each position, a vector of length *d*_*model*_ is generated. The values in even dimensions are computed using the sine function, and the values in odd dimensions are computed using the cosine function. This allows maintaining a certain relative positional relationship between vectors at different positions.

The optimization function of the Transformer model is based on Cross Entropy Loss (Ning X. et al., 2024), aiming to minimize the difference between the probability distribution of the decoder's output and the true output's probability distribution. To prevent the decoder from seeing future information when generating the next output, the Transformer model uses a masking mechanism. This involves setting the attention weights of future positions' elements in the decoder's input sequence to negative infinity, making their probability zero in the *softmax* function. Additionally, to prevent overfitting, the Transformer model employs a Dropout mechanism, randomly discarding some units or connections in the model with a certain probability. The formula for the optimization function of the Transformer model is shown in Equation (6):


(6) $$\begin{array}{l} L\left(\theta \right)=-\frac{1}{N}\overset{N}{\underset{n=1}{\sum }}\overset{T_{n}}{\underset{t=1}{\sum }}\mathrm{logp}\left(y_{t}^{n}|y_{<t}^{n},x_{2}^{n};\theta \right) \end{array}$$

In the formula, θ represents the model parameters, *N* represents the number of samples, *T*_*n*_ represents the length of the output sequence for the nth sample, *x*^*n*^ represents the input sequence for the nth sample, $y_{t}^{n}$ represents the t-th element of the output sequence for the nth sample, and $y_{t-1}^{n}$ represents the first *t*−1 elements of the output sequence for the nth sample. $𝔭\left(y_{t}^{n}|y_{<t}^{n},x_{2}^{n};0\right)$ represents the probability of the model generating the next output based on the input sequence and the previous output sequence.

In this study, we use the Transformer model to model skeletal action sequences to capture global contextual information in motion. We represent each skeletal action frame as a vector, input it into the encoder, and obtain a continuous vector representation. We use this vector representation as a query, the encoder's output as keys and values, input them into the decoder, and obtain a new vector representation used to generate the next skeletal action frame. We repeat this process until the entire skeletal action sequence is generated. Our goal is to make the generated skeletal action sequence as close as possible to the real skeletal action sequence while adhering to the physical laws and biological characteristics of motion. To achieve this goal, we use cross-entropy loss functions, masking mechanisms, positional encoding, and dropout mechanisms to optimize our model. In the next subsection, we will introduce how to use Graph Neural Networks to model local motion features for a better understanding of the relationships between joints.

### 3.2 Graph neural networks

**Figure 3:**
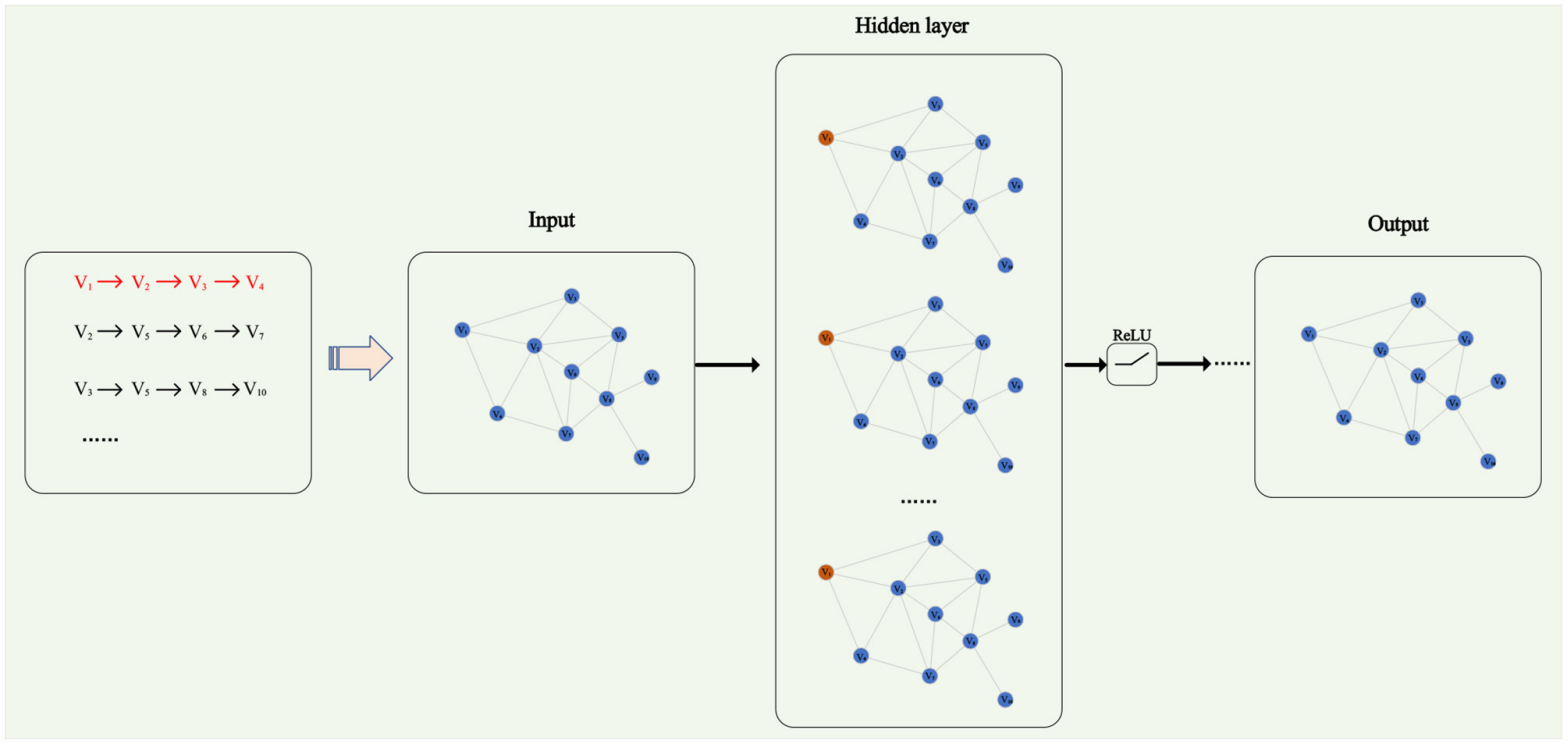
Graph neural networks.

Graph Neural Networks (GNN) is a type of artificial neural network designed to process graph-structured data (Jiang et al., 2023). Graph-structured data is a complex data type composed of nodes and edges, capable of representing various entities and relationships, such as social networks, knowledge graphs, molecular structures, etc. Zhe and Xin (2022) proposed a network structure representation learning method based on neighborhood information. This method utilizes the adjacency relationships of nodes to learn structural representations and exhibits excellent generalization capabilities. Graph Neural Networks consist of multiple graph convolutional layers and a fully connected layer. The role of the fully connected layer is to perform a non-linear transformation on the output of the graph convolutional layers, yielding the final output vector used for tasks like graph classification and node classification. The overall architecture of a Graph Neural Network is illustrated in Figure 3.

The fundamental idea of Graph Neural Networks is to enhance feature representations by enabling each node to collect and update information from neighboring nodes through a message-passing mechanism. Generally, the computation process of a Graph Neural Network can be expressed by the following formula (7) and (8):


(7) $$\begin{array}{l} h_{v}^{\left(k\right)}={\text{UPDATE}}^{\left(k\right)}\left(h_{v}^{\left(k-1\right)},{\text{AGGREGATE}}^{\left(k\right)}\left(\left\{h_{u}^{\left(k-1\right)};u\in N\left(v\right)\right\}\right)\right) \end{array}$$

(8) $$\begin{array}{l} o_{v}=\text{READOUT}\left(h_{v}^{\left(K\right)},h_{G}\right) \end{array}$$

Here, $h_{v}^{\left(k\right)}$ represents the state vector of node *v* at layer *k*, $h_{v}^{\left(0\right)}$ represents the initial feature vector of node *v*, *o*_*v*_ represents the final output vector of node *v*, *h*_*G*_ represents the global information vector of the entire graph, *N*(*v*) denotes the neighbor set of node *v*, *AGGREGATE*(*k*) represents the function for aggregating neighbor node states at layer *k*, *UPDATE*(*k*) represents the function for updating the state of the node itself at layer *k*, and *READOUT* represents the function for outputting node states and global information. These functions can be implemented differently depending on the specific Graph Neural Network model, such as using averaging, summation, maximum, concatenation, attention mechanisms, gating, and so on.

From the above formula, it can be observed that the core of a Graph Neural Network lies in the aggregation function, which determines how information from neighboring nodes is summarized for each central node. Different Graph Neural Network models employ different aggregation functions, for example:

Graph Convolutional Networks (GCNs) use a weighted average aggregation function, which is shown in Equation (9):


(9) $$\begin{array}{l} {\text{AGGREGATE}}^{\left(k\right)}\left(\left\{h_{u}^{\left(k-1\right)}:u\in N(v)\right\}\right)\\ \;=\frac{1}{|N(v)|+1}\left(h_{v}^{\left(k-1\right)}+\sum_{u\in N(v)}h_{u}^{\left(k-1\right)}\right) \end{array}$$

Graph Attention Network (GAT) uses an attention mechanism for the aggregation function, which is shown in Equation (10):


(10) $$\begin{array}{l} {\text{AGGREGATE}}^{\left(k\right)}\left(\left\{h_{u}^{\left(k-1\right)}:u\in N\left(v\right)\right\}\right)=\underset{u\in N\left(v\right)}{\sum }\alpha_{vu}h_{u}^{\left(k-1\right)} \end{array}$$

Where α_vu_ represents the attention coefficient between nodes *v* and *u*, calculated by the following formula (11):


(11) $$\begin{array}{l} \alpha_{vu}=\frac{\exp\left(\text{LeakyReLU}\left(a^{T}\left[W^{\left(k\right)}h_{v}^{\left(k-1\right)}||W^{\left(k\right)}h_{u}^{\left(k-1\right)}\right]\right)\right)}{\sum_{w\in N(v)}\exp\left(\text{LeakyReLU}\left(a^{T}\left[W^{\left(k\right)}h_{v}^{\left(k-1\right)}||W^{\left(k\right)}h_{w}^{\left(k-1\right)}\right]\right)\right)} \end{array}$$

Among them, *a* represents a learnable weight vector, and || represents a vector splicing operation. The formula of the optimization function of the graph neural network is shown in Equation (12):


(12) $$\begin{array}{l} L\left(\theta \right)=-\frac{1}{N}\overset{N}{\underset{n=1}{\sum }}\overset{C}{\underset{c=1}{\sum }}y_{n,c}\text{log}p\left(y_{n,c}|x_{n};\theta \right) \end{array}$$

Where θ represents the model parameters, *N* is the number of samples, *C* is the number of classes, *x*_*n*_ denotes the input of the nth sample, *y*_*n*_,c represents the c-th element of the true label of the nth sample, and p(y_n_, c∣x_n_; θ) represents the probability of the model predicting the c-th element of the label based on the input.

In this study, we employ a graph neural network to model each joint in the skeletal action sequence, aiming to better understand the relationships between joints. We use the position and velocity of each joint as its initial features, which are then input into the graph neural network to obtain an updated feature representation. This representation is concatenated with the output of the Transformer model, resulting in a feature representation that integrates both global and local information. This integrated representation is utilized for generating the next skeletal action frame. Our objective is to make the generated skeletal action sequence as close as possible to the real sequence while adhering to the physical laws and biological characteristics of motion. To achieve this goal, we use cross-entropy loss functions and dropout mechanisms to optimize our model. In the next subsection, we will discuss how we enhance the robustness and adaptability of the model using generative adversarial networks, making the model generate more realistic and diverse motion sequences through adversarial training.

### 3.3 Generative adversarial network

**Figure 4:**
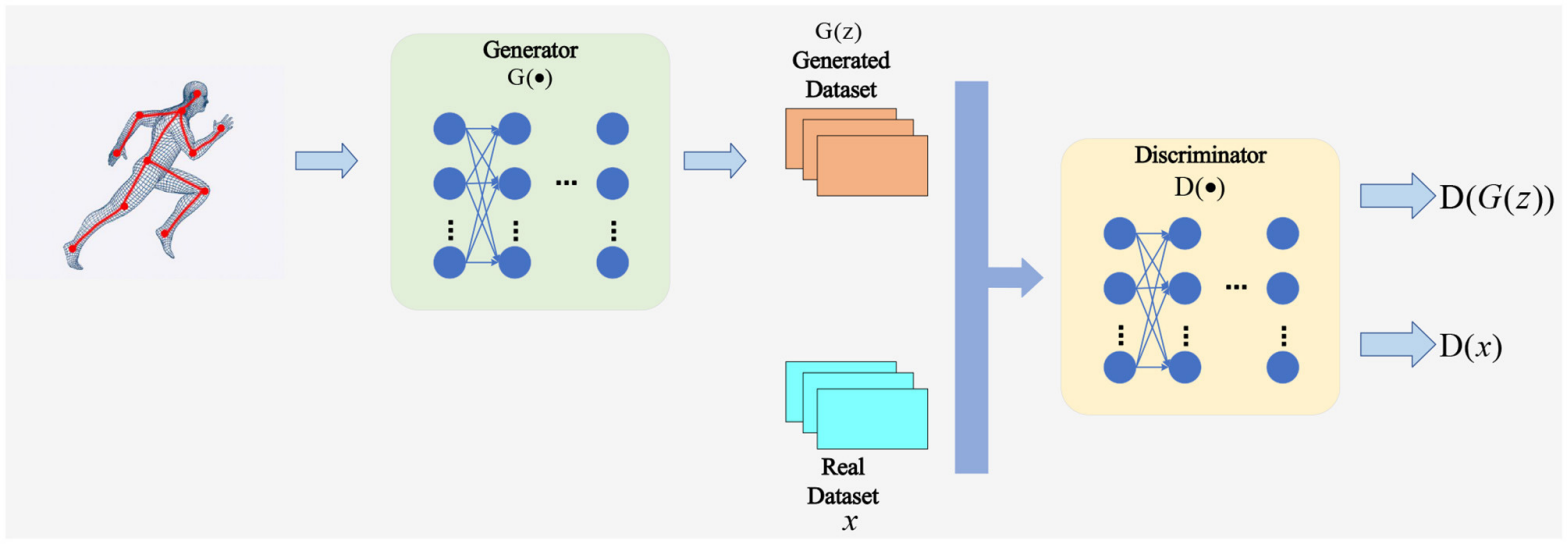
Generative adversarial network.

Generative Adversarial Network (GAN) is an unsupervised learning method that generates data through the mutual adversarial training of two neural networks—the generator (*G*) and the discriminator (*D*) (Creswell et al., 2018). In image processing tasks, generative adversarial networks have been widely applied. For example, Zhu et al. (2021) proposed a method based on generative adversarial networks to achieve single-image super-resolution reconstruction. The objective of the generator is to create data samples *G*(*z*) from a random noise vector z that resemble the real data distribution *p*_*d*_*ata*. Meanwhile, the discriminator aims to distinguish whether the input data sample x is real or generated, outputting the real probability *D*(*x*). The generator and discriminator can be viewed as two parties engaged in a zero-sum game, where the generator seeks to deceive the discriminator by making *D*(*G*(*z*)) close to 1, while the discriminator endeavors to identify the generator's forgery, pushing *D*(*G*(*z*)) close to 0. When they reach a Nash equilibrium, the generator can approximate the distribution of real data, i.e., *p*_*g*_ = *p*_*data*_. The structure of a Generative Adversarial Network model is illustrated in Figure 4.

The training process of a Generative Adversarial Network can be described by the following minimax optimization problem with Equation (13):


(13) $$\begin{array}{l} \text{minmax}V\left(D,G\right)=E_{x_{3}\sim p_{data}\left(x_{3}\right)}\left[\log D\left(x\right)\right]\\ \;+E_{z\sim p_{z}\left(z\right)}\left[\log\left(1-D\left(G\left(z\right)\right)\right)\right] \end{array}$$

This formula represents the expected log-probability output of the discriminator on a real sample *x*_3_ sampled from the true data distribution *p*_data_(*x*). Here, *E* denotes the mathematical expectation, *p*_*z*_(*z*) represents the prior distribution of noise vector *z*, typically a uniform or normal distribution. To solve this optimization problem, an alternating update strategy is commonly employed: the generator *G* is fixed while updating the discriminator *D* to maximize *V*(*D, G*).; then, the discriminator *D* is fixed while updating the generator *G* to minimize *V*(*D, G*). This process can be implemented using the following gradient descent algorithms (14) and (15):


(14) $$\begin{array}{l} \theta_{d}\leftarrow \theta_{d}+\alpha \nabla_{\theta_{d}}\frac{1}{m}\overset{m}{\underset{i=1}{\sum }}\left[\text{log}D\left(x^{\left(i\right)}\right)+\text{log}\left(1-D\left(G\left(z^{\left(i\right)}\right)\right)\right)\right] \end{array}$$

(15) $$\begin{array}{l} \theta_{g}\leftarrow \theta_{g}-\alpha \nabla_{\theta_{g}}\frac{1}{m}\overset{m}{\underset{i=1}{\sum }}\log\left(1-D\left(G\left(z^{\left(i\right)}\right)\right)\right) \end{array}$$

where θ_*d*_ and θ_*g*_ represent the parameters of the discriminator and generator, respectively. α is the learning rate, *m* is the batch size, *x*^(*i*)^ and *z*^(*i*)^ represent the *i*-th real data sample and noise vector.

An important advantage of Generative Adversarial Networks (GANs) is that they do not require any annotated data; instead, they can learn intrinsic features from a large amount of unlabeled data and generate new data samples. GANs can also be combined with other deep learning models such as Convolutional Neural Networks (CNNs), Recurrent Neural Networks (RNNs), Variational Autoencoders (VAEs), etc., to play roles in various domains and tasks, including image generation, text generation, speech generation, etc.

In this study, we use GANs to enhance the robustness and adaptability of our skeleton action sequence generation model. We design the generator as a deep neural network based on Transformer and Graph Neural Network, while the discriminator is designed as a binary classifier based on Convolutional Neural Network. Our goal is to make the generator produce more realistic and diverse skeleton action sequences, thereby improving the effectiveness of motion training and injury prevention. To achieve this goal, we use the following GAN loss function formulation (16):


(16) $$\begin{array}{l} L_{GAN}\left(G,D\right)=E_{x\sim p_{data}\left(x\right)}\left[\log D\left(x\right)\right]+E_{z\sim p_{z}\left(z\right)}\left[\log\left(1-D\left(G\left(z\right)\right)\right)\right]\\ \;+\lambda E_{x\sim p_{data}\left(x\right)}\left[{\left(D\left(x\right)-1\right)}^{2}\right] \end{array}$$

where λ is a regularization coefficient used to penalize the misjudgment of the discriminator on real data, thereby enhancing the discriminator's discriminative ability and preventing the generator from converging too early to a local optimum.

In this chapter, we introduced our skeleton action analysis method, including three main components: the Transformer model, Graph Neural Network, and Generative Adversarial Network. The Transformer model is used for global modeling of skeleton action sequences, capturing long-term dependencies and contextual information in motion. The Graph Neural Network is used for local modeling of skeleton action sequences, capturing spatial relationships and motion characteristics between joints. The Generative Adversarial Network is employed to enhance the authenticity and diversity of skeleton action sequences. Through adversarial training, the generator produces motion sequences that better conform to the distribution of real data. Our method combines these three advanced deep learning technologies to achieve the goal of optimizing motion training and preventing injuries. In the next chapter, we will present our experimental setup and results analysis, demonstrating the performance of our method on different datasets and evaluation metrics.

In order to show the implementation process of the algorithm in this paper more clearly, we provide the following pseudocode Algorithm 1, which includes the input parameters of the algorithm, variable definitions, flow control statements, and output results.

**Algorithm 1:**
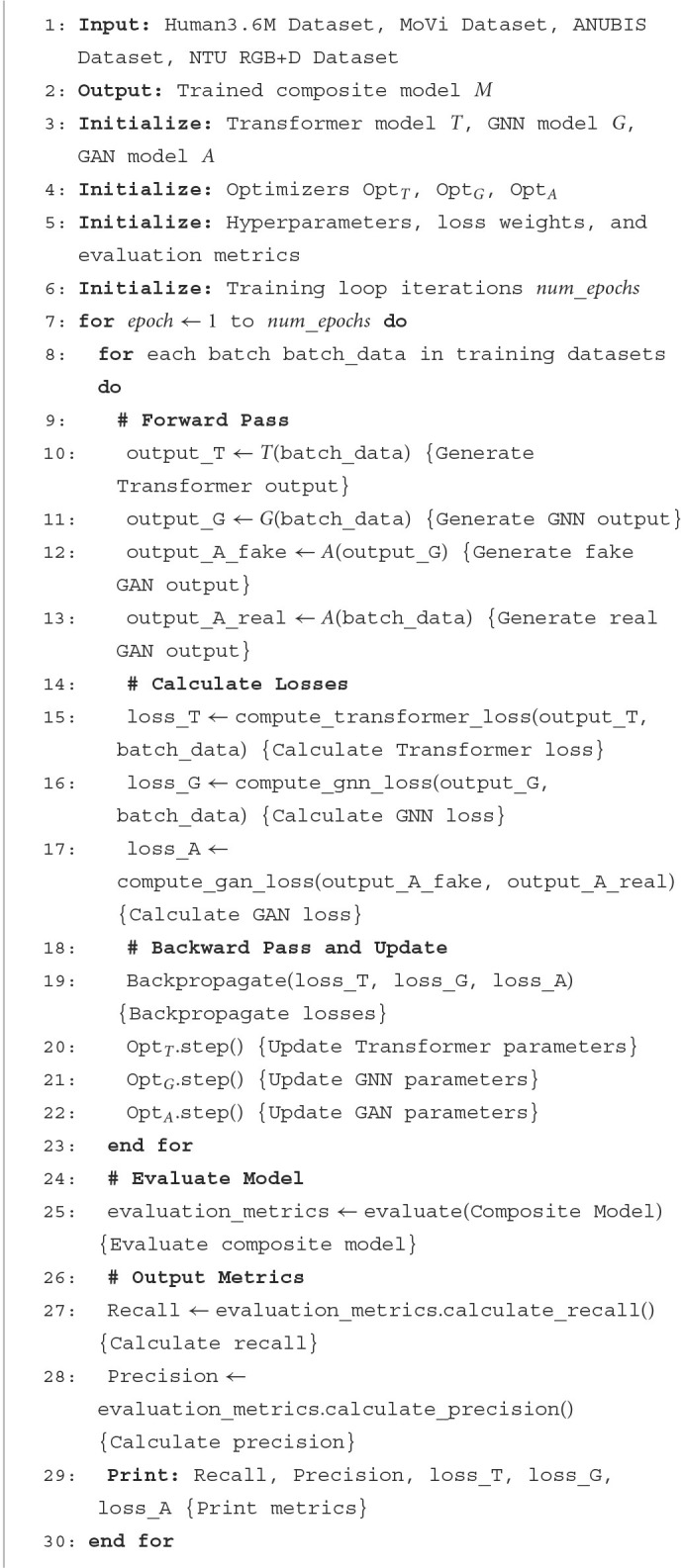
Training composite model.

### 3.4 Algorithm process description

1. Input data: The algorithm takes four datasets as input, namely the Human3.6M Dataset, MoVi Dataset, ANUBIS Dataset, and NTU RGB+D Dataset, which contain relevant information about skeletal motion.
2. Initialization: At the beginning of the algorithm, three models are initialized: the Transformer model *T*, the Graph Neural Network model *G*, and the Generative Adversarial Network model *A*. Corresponding optimizers Opt_*T*_, Opt_*G*_, Opt_*A*_, hyperparameters, loss weights, and evaluation metrics are also initialized. The number of epochs for the training loop num_epochs is set.
3. Training loop: For each training epoch, the algorithm iterates over each training data batch batch_data.
4. Forward propagation: For each training batch, forward propagation is performed. The batch data is inputted into the Transformer model, GNN model, and GAN model, generating Transformer output output_T, GNN output output_G, and GAN-generated fake output output_A_fake as well as real output output_A_real.
5. Compute loss: After forward propagation, the loss of the Transformer model, GNN model, and GAN model is computed. The loss of the Transformer model is calculated by the compute_transformer_loss function, the loss of the GNN model is calculated by the compute_gnn_loss function, and the loss of the GAN model is calculated by the compute_gan_loss function.
6. Backward propagation and update: After computing the loss, backward propagation is performed, and model parameters are updated based on the gradients obtained from backward propagation. The parameters are updated using optimizers Opt_*T*_, Opt_*G*_, Opt_*A*_.
7. Evaluate model: At the end of each training epoch, the trained composite model is evaluated, and performance metrics are calculated.
8. Output metrics: Output performance metrics for each training epoch, including Recall, Precision, Transformer model loss, GNN model loss, and GAN model loss.

A. Data transfer and computational details between modules

- Data transfer: Data is transferred from the input datasets to the Transformer model, GNN model, and GAN model. This is done through forward propagation to obtain outputs from each model, followed by loss computation and parameter updates through backward propagation.
- Computational details:
  - The transformer model receives input data and models it using self-attention mechanisms to generate Transformer output.
  - The GNN model receives input data and models local motion features using graph neural networks to generate GNN output.
  - The GAN model receives output from the GNN model and generates fake motion sequences using generative adversarial networks. It also receives real motion sequences and computes the loss for the generative adversarial network.
  - Loss functions are computed to measure the performance of the models based on the differences between the outputs of different models and the actual data.
  - Backward propagation involves computing gradients of the loss function and propagating gradient information back to each model. Model parameters are updated using optimizers.

B. Potential randomness or uncertainty factors

- Randomness in data batches: Each training batch during the training process may be randomly sampled. Therefore, the specific data flow and model parameter updates may vary for each training epoch.
- Randomness in parameter initialization: Model parameters may be initialized using random initialization methods. Thus, the initial state of model parameters may differ each time training begins, which can impact the final training results.

## 4 Experiment

**Figure 5:**
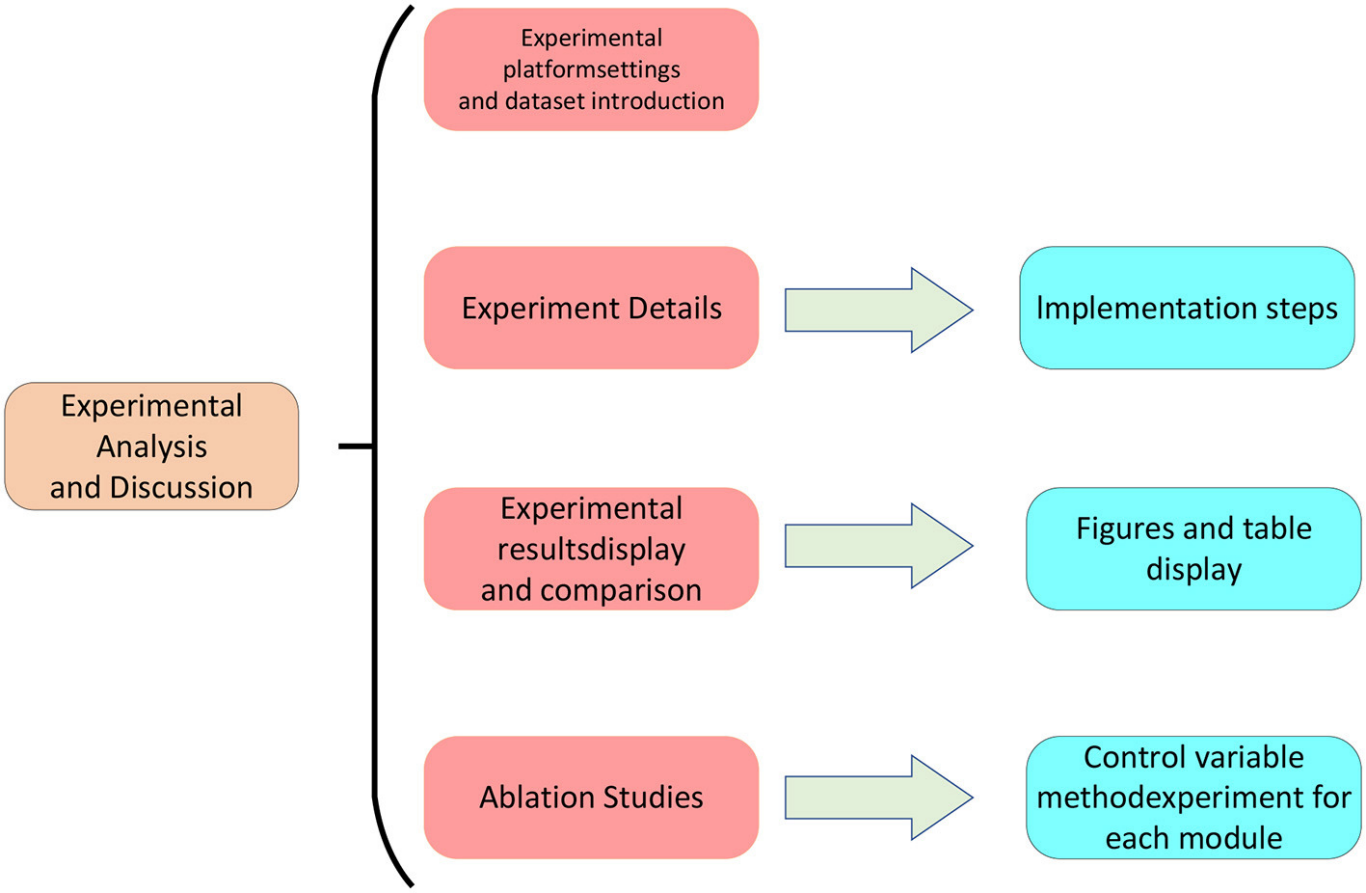
Experimental flow chart.

In the previous chapter, we introduced our skeleton action analysis method, which consists of three main components: the Transformer model, Graph Neural Network, and Generative Adversarial Network. In this chapter, we will present our experimental setup and results analysis, showcasing the performance of our method on different datasets and evaluation metrics. We will begin by introducing our experimental environment, including hardware configuration and software platforms. Next, we will describe the datasets we used, covering data sources, scale, and other relevant details. Subsequently, we will introduce the evaluation metrics employed, including specificity, accuracy, recall, and *F*1-score, along with their calculation formulas and meanings. Finally, we will conduct a data analysis of our experimental results, including comparisons with other methods, the impact of different model parameters, and the adaptability to various datasets. The overall flowchart of this experiment is depicted in Figure 5.

### 4.1 Experimental environment

- Hardware environment
  This experiment utilized an advanced computing server equipped with an AMD Ryzen 9 5950X 16-Core Processor @ 3.40 GHz CPU and 256 GB RAM, featuring four Nvidia GeForce RTX 3080 16 GB GPUs. This hardware configuration provided outstanding computing and memory resources, significantly facilitating the efficient training and inference of deep learning tasks. Such robust hardware capabilities contributed to accelerating the model training process, ensuring the experiment smoothly operated in a high-performance computing environment, thereby enhancing efficiency and reliability.
- Software environment
  Python served as the primary programming language for this experiment, with PyTorch employed as the deep learning framework to construct a skeleton action analysis model based on Transformer and Graph Neural Network architectures. PyTorch offered concise and efficient interfaces for model construction and training, enabling flexible design and optimization of the skeleton action analysis model. Leveraging PyTorch's parallel computing and automatic differentiation features, we effectively accelerated the model training speed, ensuring rapid convergence and outstanding performance. The collaborative use of Python and PyTorch provided robust and convenient software support for our research, laying a solid technical foundation for the study and experimentation of skeleton action analysis methods.

### 4.2 Experimental data

- Human3.6M Dataset
  The Human3.6M dataset is a large publicly available dataset designed for research in 3D human pose estimation. Proposed by Catalin Ionescu and colleagues at the Institute of Mathematics and Computer Science (IMAR) in Romania in 2014, this dataset comprises ~3.6 million 3D human poses paired with corresponding images. The data collection involved 11 professional actors performing various activities across 17 different scenes, including discussions, smoking, taking photos, making phone calls, and more. The data collection process utilized a high-speed motion capture system and four synchronized high-resolution cameras, capturing video data at a rate of 50 frames per second. The dataset not only provides precise 3D joint positions and joint angles but also includes pixel-level labels for 24 body parts of each human pose. Additional data such as time-of-flight range data, 3D laser scan data of actors, accurate background segmentation, and bounding boxes around individuals are also available. Precomputed image descriptors, visualizations, and software for discriminative human pose prediction are included, along with a reserved test set for performance evaluation. The Human3.6M dataset stands as the most extensive and widely used dataset in the field of 3D human pose estimation. It spans various tasks related to 3D human pose, including 3D human pose estimation, video prediction, human motion generation, human body part segmentation, and human pose retrieval. This dataset holds significant importance and value for researching methods in 3D human motion analysis in natural environments.
- MoVi Dataset
  The MoVi dataset is a large, versatile dataset encompassing human motion and video data, released in 2021 by the BioMotionLab at York University, Canada. This dataset includes 60 female and 30 male actors who perform 20 predefined daily and exercise-related actions, along with one self-selected action. The actions span a variety of scenarios such as walking, running, jumping, dancing, playing sports, boxing, cycling, and more. Notably, the dataset provides synchronized data on poses, body grids, and video recordings, making it applicable to multiple domains, including human pose estimation and tracking, human motion prediction and synthesis, action recognition, and gait analysis. The distinctive feature of this dataset lies in its simultaneous provision of synchronized poses, body grids, and video recordings, facilitating applications in human pose estimation and tracking, human motion prediction and synthesis, action recognition, and gait analysis across various domains. This dataset holds significant value for our research as it offers rich human motion data for training and testing our methods. We leverage the MoVi dataset's pose, body grid, and video data to extract motion features, evaluate motion quality, generate motion suggestions, and predict motion injuries.
- ANUBIS Dataset
  The ANUBIS Dataset is a large-scale 3D skeleton action recognition dataset collected and released in 2022 by researchers from the College of Engineering and Computer Science at the Australian National University. This dataset employs Azure-Kinect cameras to capture 80 different human actions, spanning daily activities, sports, social interactions, bullying, and scenarios related to the COVID-19 pandemic. Each action is performed by multiple subjects in various environments and is captured from both frontal and rear perspectives. Each skeleton action frame includes position and velocity information for 32 human joints, along with corresponding depth and RGB images.The ANUBIS Dataset offers several advantages compared to previous skeleton action recognition datasets: 1. Advanced Sensors: Enhanced data quality and accuracy are achieved through the use of more advanced sensors. 2. Novel Rear-view Perspective: The inclusion of a novel rear-view perspective adds diversity and complexity to the dataset. 3. Encouragement of Natural Movement: Emphasis on subjects' enthusiasm and naturalness enhances the realism and credibility of the data. 4. Inclusion of COVID-19 Era Actions: The dataset includes actions reflecting the COVID-19 era, demonstrating timeliness and societal relevance.The ANUBIS Dataset holds significant value for our research as it provides a rich, multi-perspective, and high-quality source of skeleton action data for training and testing our models.
- NTU RGB+D Dataset
  The NTU RGB+D Dataset is a large-scale RGB-D human action recognition dataset introduced by Shahroudy et al. from Nanyang Technological University at the CVPR conference in 2016. The dataset comprises 56,880 action samples covering 60 action categories performed by 40 different individuals. Actions are categorized into three main classes: 40 daily activities (e.g., drinking, eating, reading), nine health-related actions (e.g., sneezing, shaking, falling), and 11 interactive actions (e.g., boxing, kicking, hugging). These actions are performed under 17 distinct environmental conditions, corresponding to 17 video sequences (S0010-S017).Captured using three Microsoft Kinect V2 cameras simultaneously from different horizontal perspectives (−45 degrees, 0 degrees, and +45 degrees), each sample provides four modalities of information: RGB videos, depth map sequences, 3D skeleton data, and infrared videos. Performance evaluation for action recognition includes cross-subject testing and cross-view testing. Cross-subject testing involves splitting the 40 individuals into training and testing groups, while cross-view testing uses one camera (+45 degrees) for testing and the other two for training. In summary, the NTU RGB+D Dataset offers a rich, diverse, and high-quality RGB-D human action data source for research in action recognition.

### 4.3 Evaluation index

To comprehensively assess the performance of our method in skeleton action analysis, we employed several metrics to measure the effectiveness of our model. These metrics include specificity, accuracy, recall, and *F*1-score. Specificity represents the proportion of correctly classifying negative samples (i.e., incorrect actions) as negative, reflecting the discriminative capability of the model. Accuracy measures the proportion of correctly classifying both positive samples (i.e., correct actions) and negative samples, showcasing the model's overall correctness. Recall indicates the proportion of correctly classifying positive samples as positive, highlighting the model's coverage ability. *F*1-score is the harmonic mean of accuracy and recall, providing a comprehensive assessment of the model's performance. In the following sections, we will provide a detailed overview of our method's performance on these metrics and compare it with other approaches.

- Specificity
  Specificity is a crucial evaluation metric that measures the model's performance on negative instances, i.e., its ability to correctly predict negatives. In skeleton action analysis, specificity helps us understand the model's recognition accuracy for non-target actions, providing a more comprehensive assessment of its practical utility.
- The formula for specificity is shown in (17).
(17) $$\begin{array}{l} \text{Specificity}=\frac{\text{True Negatives}}{\text{True Negatives}+\text{False Positives}}\times 100\% \end{array}$$
  In this context, the parameters are interpreted as follows: True Negatives (TN) represent the number of negative instances correctly predicted by the model, i.e., the actual negatives correctly classified as negatives. False Positives (FP) denote the number of negative instances incorrectly predicted as positives, i.e., the actual negatives incorrectly classified as positives.
  The percentage value of specificity indicates the model's success in recognizing negatives among all actual negative instances. In skeleton action analysis, a high specificity value suggests that the model excels in discerning non-target actions, helping avoid misclassifying normal actions as target actions and thereby enhancing the model's practical utility.
  Through specificity calculation, we gain a comprehensive understanding of the model's performance in handling negative instances, providing essential insights into the evaluation of our skeleton action analysis method's performance. Specificity, along with other evaluation metrics, will be presented in the paper to comprehensively showcase the proposed method's overall performance in optimizing motion training and preventing injuries.
- Accuracy
  Accuracy is a fundamental metric for assessing the overall performance of a model across all categories, measuring the proportion of correctly predicted samples relative to the total number of samples. In skeleton action analysis, accuracy serves as a crucial criterion for evaluating the model's global performance, providing a direct reflection of its overall effectiveness in the task. The formula for calculating accuracy is shown in (18).
(18) $$\begin{array}{l} \text{Accuracy}=\frac{\text{True Positives}+\text{True Negatives}}{\text{Total Samples}}\times 100\% \end{array}$$
  Where each parameter is explained as follows: True Positives (TP) represent the number of positive instances correctly predicted by the model, i.e., the number of actual positives correctly classified as positives. True Negatives (TN) represent the number of negative instances correctly predicted by the model, i.e., the number of actual negatives correctly classified as negatives. Total Samples denote the total number of samples, including both positives and negatives.
  The percentage value of accuracy reflects the overall correctness of the model across the entire dataset. In the task of skeleton action analysis, a high accuracy indicates that the model exhibits strong classification capabilities for both positive and negative samples, effectively recognizing the target skeleton actions and providing reliable support for optimizing motion training and preventing injuries.
  In our study, accuracy will serve as a core evaluation metric, presented alongside other metrics to comprehensively assess the performance of the proposed skeleton action analysis method based on Transformer and graph neural networks. A high accuracy value will reinforce the feasibility and effectiveness of our method in practical applications.
- Recall
  Recall is a crucial metric for assessing the model's performance on positive instances. It measures the model's ability to correctly predict positives, indicating the extent to which the model can capture actual positive samples. In skeleton action analysis, the recall directly correlates with the model's effectiveness in recognizing and capturing target actions. The formula for recall is shown in (19).
(19) $$\begin{array}{l} \text{Recall}=\frac{\text{True Positives}}{\text{True Positives}+\text{False Negatives}}\times 100\% \end{array}$$
  Whereas, the parameters are explained as follows: True Positives (TP) represent the number of positive instances correctly predicted by the model, i.e., the actual positives correctly classified as positives. False Negatives (FN) indicate the number of instances where the model incorrectly predicted negatives, i.e., actual positives incorrectly classified as negatives.
  The percentage value of recall signifies the model's success in identifying positives among all actual positive instances. In skeleton action analysis tasks, a high recall implies that the model can effectively capture target actions, reducing the risk of misclassifying true positives as negatives. This is crucial for enhancing the precision of motion training and the effectiveness of injury prevention.
  In our study, recall will serve as a key metric, providing insights into the model's capability to identify positive instances. We will present a comprehensive view of the performance of the Transformer and graph neural network-based skeleton action analysis method in experiments, considering other evaluation metrics.
- *F*1-score
  *F*1-score is a comprehensive metric that evaluates the precision and recall of a model by harmonizing these two aspects to balance the model's overall performance and accuracy. In skeleton action analysis, *F*1-score is a crucial performance measure, particularly useful for handling imbalanced datasets. The formula for calculating *F*1-score is shown in (20).
(20) $$\begin{array}{l} \text{F1-score}=\frac{2\times \text{Precision}\times \text{Recall}}{\text{Precision}+\text{Recall}}\times 100\% \end{array}$$
  Whereas, the parameters are explained as follows: Precision is used to measure the accuracy of the model in positive predictions, representing the proportion of correctly predicted positive instances among all samples predicted as positive. Recall gauges the model's ability to capture actual positive instances, indicating the proportion of correctly predicted positive instances among all actual positives.
  F1-score, by considering both Precision and Recall, aims to find a balance suitable for situations with significant differences in sample quantities between different classes. In skeleton action analysis, a high F1-score indicates that the model has achieved a good balance between comprehensiveness and precision, crucial for ensuring the overall performance of the model in recognizing target actions.
  In our study, *F*1-score will be presented alongside other evaluation metrics, providing a comprehensive perspective for a thorough assessment of our proposed skeleton action analysis method based on Transformer and graph neural networks in the experiments.

### 4.4 Experimental comparison and analysis

After an in-depth investigation into the performance of skeleton action analysis methods, our focus will shift to a comparative analysis between the proposed method based on Transformer and graph neural networks and traditional approaches. Through meticulous experimental design and comprehensive performance evaluation, our aim is to unveil the superiority of the proposed method in optimizing motion training and preventing injuries. We will compare traditional skeleton action analysis methods, exploring their performance on key metrics such as specificity, accuracy, recall, and *F*1-score. This will contribute to an intuitive understanding of the advantages of the proposed method over traditional approaches, providing empirical support for our research. Simultaneously, we will delve into the analysis of experimental results, paying attention to performance variations across different categories and scenarios. Through detailed analysis, we can reveal the adaptability and generalization capabilities of the proposed method for different skeleton actions, further highlighting its potential applications in real-world motion scenarios.

**Figure 6:**
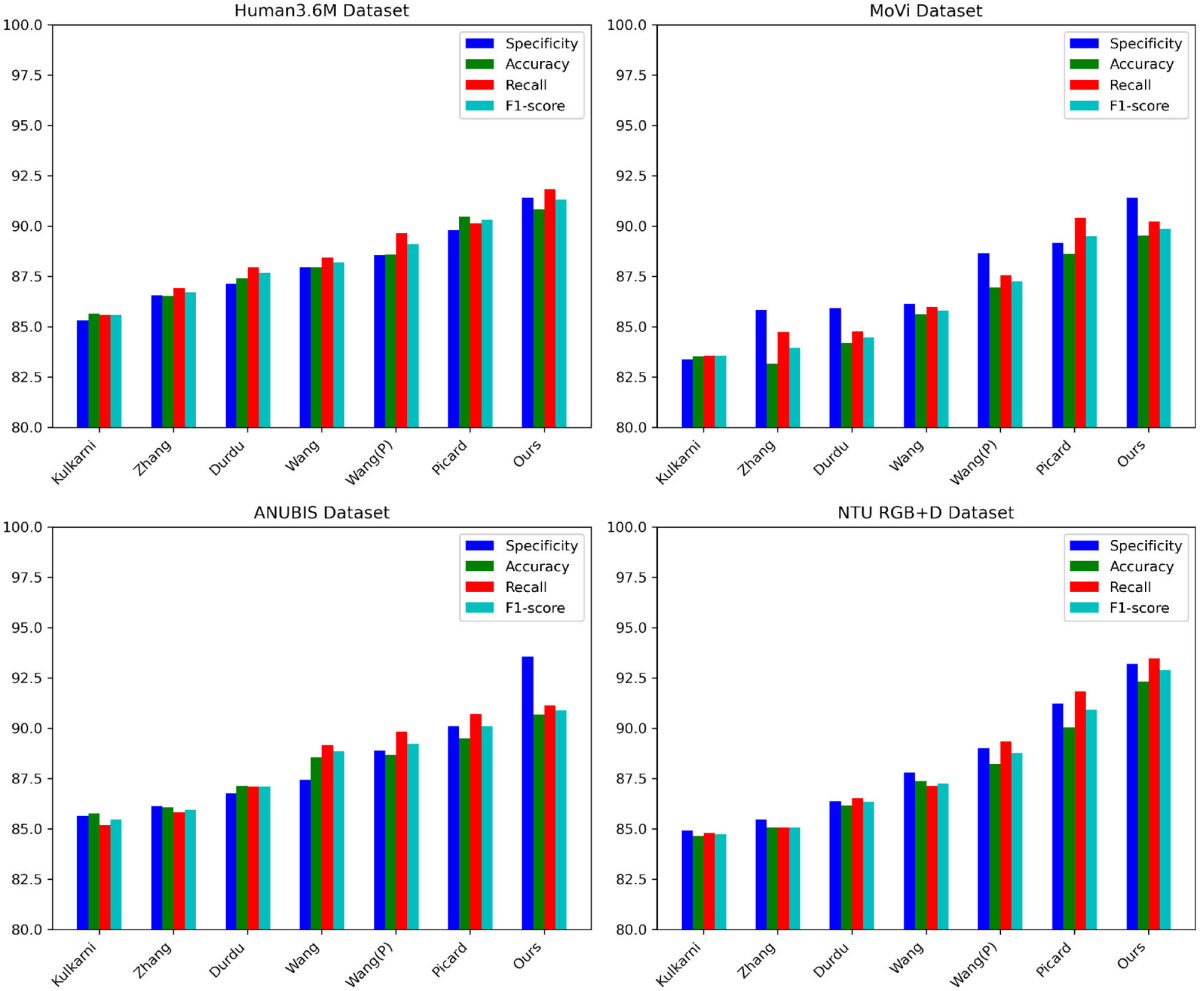
Comparative visualization of specificity, accuracy, recall, and *F*1-score indicators in different methods based on four data sets.

The data from Tables 1, 2 indicate that our proposed model outperforms other state-of-the-art approaches on key metrics. On the Human3.6M dataset, our model demonstrates comprehensive superiority over the method proposed by Wang et al., with specificity surpassing by ~3%, and accuracy and recall rates exceeding by nearly 1 percentage point. Additionally, our precision and recall rates outperform the second-ranked approach by Picard et al., achieving a 1% higher F1-score compared to Picard et al.'s method. On the MoVi dataset, our model exhibits an improvement of nearly 8% in specificity and around 7% in recall compared to Kulkarni et al.'s method, similarly outperforming Picard et al.'s approach. Furthermore, on the ANUBIS dataset, our model's specificity surpasses Picard's method by nearly 4 percentage points, with a recall rate improvement of almost 2%, showcasing outstanding performance. For the NTU RGB+D dataset, our model achieves metrics of over 91% across the board, surpassing Wang et al.'s method by over 3% on various indicators. Overall, our newly proposed framework maximizes the advantages of Graph Neural Networks and Generative Adversarial Networks, enhancing the model's learning and generalization capabilities by leveraging both spatial and temporal information. Compared to previous methods, our model consistently performs at a top-tier level across these four representative human action recognition tasks, generally surpassing recent peer works by 1–5 percentage points on key evaluation metrics. This robustly validates the significance and potential of our research efforts. Finally, we visualize the data results from Tables 1, 2 in the figures shown as Figure 6.

**Table 1:** Comparison of specificity, accuracy, recall, and *F*1-score indicators in different methods based on Human3.6M and MoVi data sets.

**Model**	**Datasets**							
	**Human3.6M Dataset (Ionescu et al.**, **2013****)**				**MoVi Dataset (Ghorbani et al.**, **2020****)**			
	**Specificity (%)**	**Accuracy (%)**	**Recall (%)**	***F*** **1-score**	**Specificity (%)**	**Accuracy (%)**	**Recall (%)**	***F*** **1-score**
Kulkarni et al. (2020)	85.31	85.63	85.60	85.61	83.37	83.53	83.55	83.54
Zhang et al. (2021)	86.55	86.51	86.94	86.72	85.84	83.16	84.73	83.94
Aslan et al. (2020)	87.14	87.39	87.97	87.68	85.93	84.19	84.76	84.47
Wang et al. (2019)	87.97	87.96	88.44	88.20	86.15	85.62	85.97	85.79
Wang et al. (2018)	88.56	88.58	89.65	89.11	88.64	86.95	87.54	87.24
Luvizon et al. (2018)	89.81	90.49	90.14	90.31	89.17	88.63	90.40	89.51
Ours	91.39	90.83	91.84	91.33	91.42	89.52	90.22	89.87

**Table 2:** Comparison of specificity, accuracy, recall, and *F*1-score indicators in different methods based on ANUBIS and NTU RGB+D data sets.

**Model**	**Datasets**							
		**ANUBIS Dataset (Qin et al.**, **2022****)**				**NTU RGB+D Dataset (Shahroudy et al.**, **2016****)**			
		**Specificity (%)**	**Accuracy (%)**	**Recall (%)**	***F*** **1-score**	**Specificity (%)**	**Accuracy (%)**	**Recall (%)**	***F*** **1-score**
Kulkarni et al. (2020)	85.66	85.77	85.19	85.48	84.94	84.65	84.81	84.73
Zhang et al. (2021)	86.14	86.06	85.84	85.95	85.47	85.07	85.08	85.07
Aslan et al. (2020)	86.78	87.14	87.11	87.12	86.38	86.19	86.54	86.36
Wang et al. (2019)	87.45	88.56	89.18	88.87	87.80	87.38	87.13	87.25
Wang et al. (2018)	88.91	88.68	89.83	89.25	89.01	88.24	89.34	88.79
Luvizon et al. (2018)	90.13	89.50	90.71	90.10	91.24	90.05	91.85	90.94
Ours	93.57	90.69	91.16	90.92	93.19	92.34	93.49	92.91

**Figure 7:**
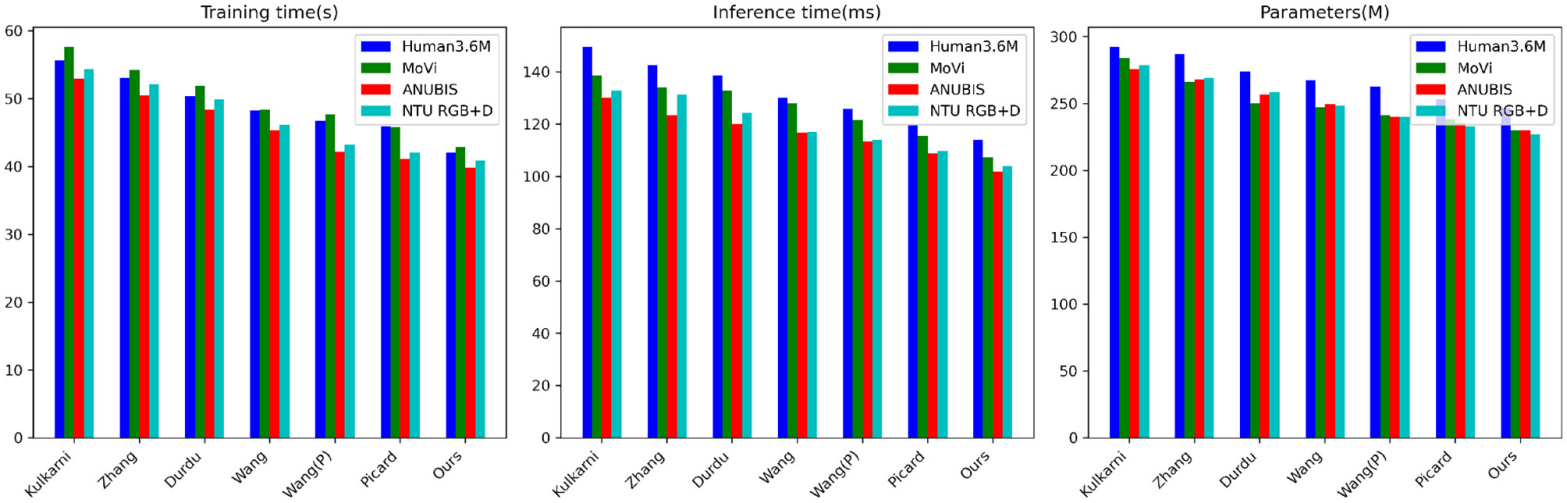
Visualization of comparison of training time, inference time, and parameters indicators in different methods based on four data sets.

From the data in Tables 3, 4, it is evident that our proposed model outperforms peer methods in several metrics, including training time, inference time, and model complexity. Taking the Human3.6M dataset as an example, our model exhibits a training time shorter by ~4 s compared to the best-performing Picard method, with an inference time nearly 7 milliseconds faster and a reduction of almost 7 million parameters. On the MoVi dataset, our model's training time is ~6 s faster than the Wang method, with an inference time improvement exceeding 14 ms and a reduction of nearly 11 million parameters. Furthermore, on the ANUBIS and NTU RGB+D datasets, our model demonstrates even more significant advantages across all metrics. Particularly on ANUBIS, the training time is almost 8 s faster than the Durdu method, the inference time is nearly 19 ms faster, and the parameter count is reduced by over 26 million. This strongly highlights the outstanding performance of our architecture in terms of learning efficiency and real-time capabilities. Overall, compared to previous works with classical structures, our design combining a new framework significantly reduces model training and inference times while ensuring accuracy. Additionally, it achieves substantial parameter compression. The core contribution of these experimental results creates better conditions for practical applications. Similarly, we have visualized the data results from Tables 3, 4 in Figure 7.

**Table 3:** Comparison of training time, inference time, and parameters indicators in different methods based on Human3.6M and MoVi data sets.

**Model**	**Datasets**					
		**Human3.6M Dataset (Ionescu et al.**, **2013****)**			**MoVi Dataset (Ghorbani et al.**, **2020****)**		
		**Training time (s)**	**Inference time (ms)**	**Parameters (M)**	**Training time (s)**	**Inference time (ms)**	**Parameters (M)**
Kulkarni et al. (2020)	55.65	149.58	292.42	57.66	138.57	284.37
Zhang et al. (2021)	53.15	142.67	287.16	54.21	134.09	266.17
Aslan et al. (2020)	50.38	138.47	273.94	51.94	132.84	250.45
Wang et al. (2019)	48.27	130.11	267.57	48.37	127.96	246.91
Wang et al. (2018)	46.79	125.93	262.73	47.69	121.80	241.56
Luvizon et al. (2018)	45.96	120.08	253.14	45.81	115.69	238.49
Ours	42.08	113.95	246.64	42.90	107.40	230.04

**Table 4:** Comparison of training time, inference time, and parameters indicators in different methods based on ANUBIS and NTU RGB+D data sets.

**Model**	**Datasets**					
		**ANUBIS Dataset (Qin et al.**, **2022****)**			**NTU RGB+D Dataset (Shahroudy et al.**, **2016****)**		
		**Training time (s)**	**Inference time (ms)**	**Parameters (M)**	**Training time (s)**	**Inference time (ms)**	**Parameters (M)**
Kulkarni et al. (2020)	52.92	129.96	275.74	54.32	132.76	278.61
Zhang et al. (2021)	50.55	123.34	268.19	52.15	131.15	269.12
Aslan et al. (2020)	48.44	120.14	256.47	49.93	124.39	258.41
Wang et al. (2019)	45.37	116.92	249.67	46.17	117.08	248.33
Wang et al. (2018)	42.16	113.55	240.01	43.29	113.95	239.79
Luvizon et al. (2018)	41.12	108.71	234.96	42.07	109.87	233.13
Ours	39.87	101.75	229.87	40.89	103.88	227.19

**Figure 8:**
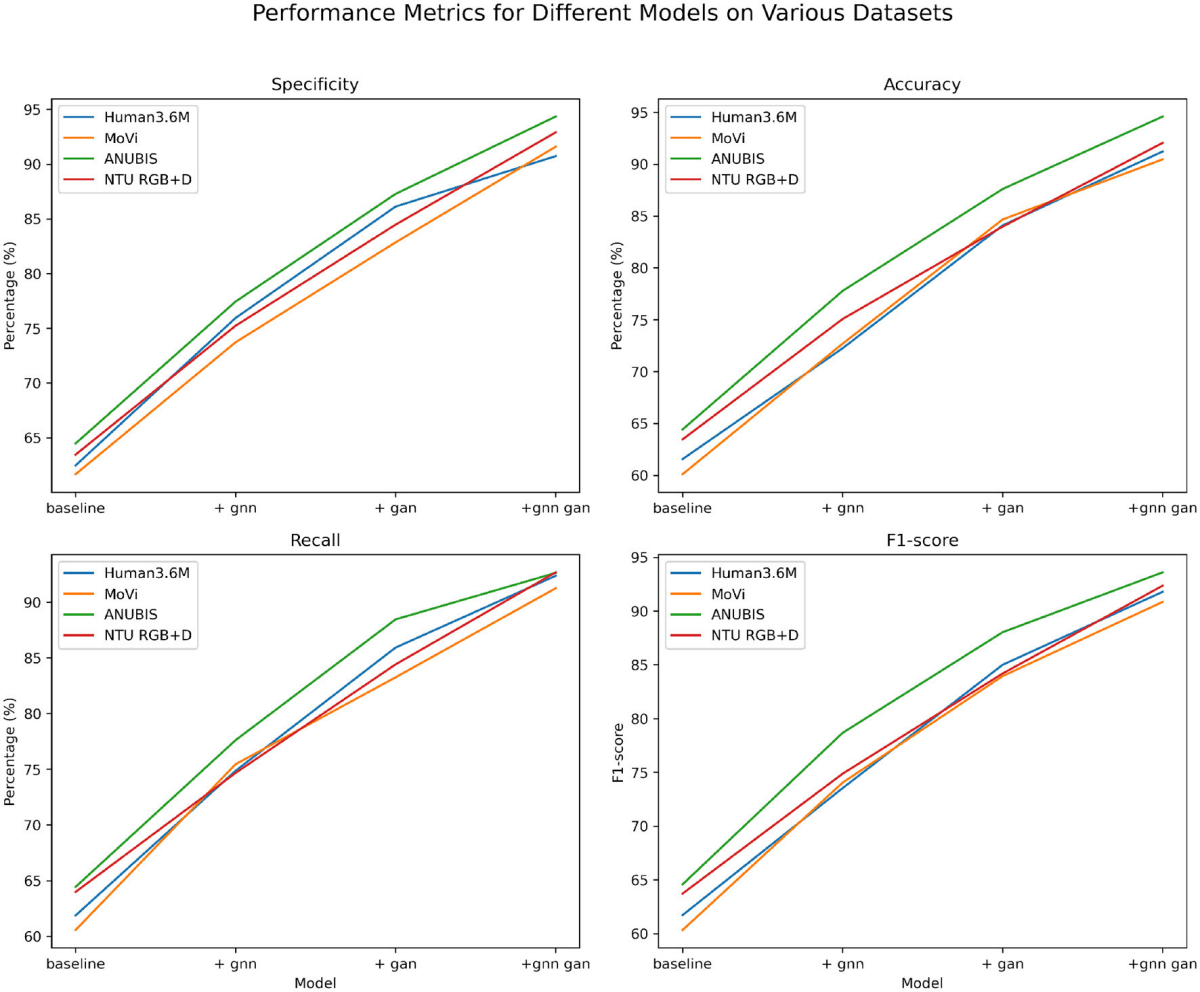
Comparative visualization of specificity, accuracy, recall, and *F*1-score indicators based on four data sets under different modules.

The data from Tables 5, 6 reveals a significant improvement in performance metrics as the model structure evolves. The simple baseline structure generally hovers around 60%. After incorporating the GNN module, there is a substantial improvement in key metrics, with recall increasing by nearly 15%. With the addition of GAN, all metrics further optimize, with specificity and accuracy improving by over 10%. When adopting the joint learning framework combining GNN and GAN, the metrics outperform other structures comprehensively across all datasets. For instance, on ANUBIS, specificity increases by almost 30%, and accuracy also improves by 30 percentage points. On the NTU RGB+D dataset, our model achieves metrics of nearly 92% across the board, surpassing the performance of using individual modules. This demonstrates the synergistic effect of the two technologies significantly enhancing model performance. Overall, as the model design continues to be optimized and upgraded, from the basic baseline to incorporating GNN and GAN separately, and then to our proposed joint end-to-end learning, the human action recognition capabilities across datasets continue to improve. The primary evaluation metrics show a comprehensive enhancement, reflecting the significant contribution of this work. Additionally, we have visualized the data results from Tables 5, 6 in Figure 8.

**Table 5:** Comparison of specificity, accuracy, recall, and *F*1-score indicators under different modules based on Human3.6M and MoVi data sets.

**Model**	**Datasets**							
	**Human3.6M Dataset (Ionescu et al.**, **2013****)**				**MoVi Dataset (Ghorbani et al.**, **2020****)**			
	**Specificity (%)**	**Accuracy (%)**	**Recall (%)**	***F*** **1-score**	**Specificity (%)**	**Accuracy (%)**	**Recall (%)**	***F*** **1-score**
Baseline	62.47	61.57	61.89	61.73	61.67	60.11	60.58	60.34
+ gnn	75.95	72.26	74.87	73.54	73.73	72.70	75.46	74.05
+ gan	86.13	84.11	85.93	85.01	82.86	84.68	83.26	83.96
+gnn gan	90.74	91.24	92.37	91.80	91.60	90.48	91.25	90.86

**Table 6:** Comparison of specificity, accuracy, recall, and *F*1-score indicators under different modules based on ANUBIS and NTU RGB+D data sets.

**Model**	**Datasets**							
		**ANUBIS Dataset (Qin et al.**, **2022****)**				**NTU RGB+D Dataset (Shahroudy et al.**, **2016****)**			
		**Specificity (%)**	**Accuracy (%)**	**Recall (%)**	***F*** **1-score**	**Specificity (%)**	**Accuracy (%)**	**Recall (%)**	***F*** **1-score**
Baseline	64.48	64.43	64.45	64.59	63.46	63.47	64.00	63.73
+ gnn	77.45	77.79	77.62	78.68	75.24	75.08	74.68	74.88
+ gan	87.29	87.62	88.46	88.04	84.48	83.99	84.42	84.20
+gnn gan	94.36	94.61	92.63	93.61	92.91	92.07	92.68	92.37

**Figure 9:**
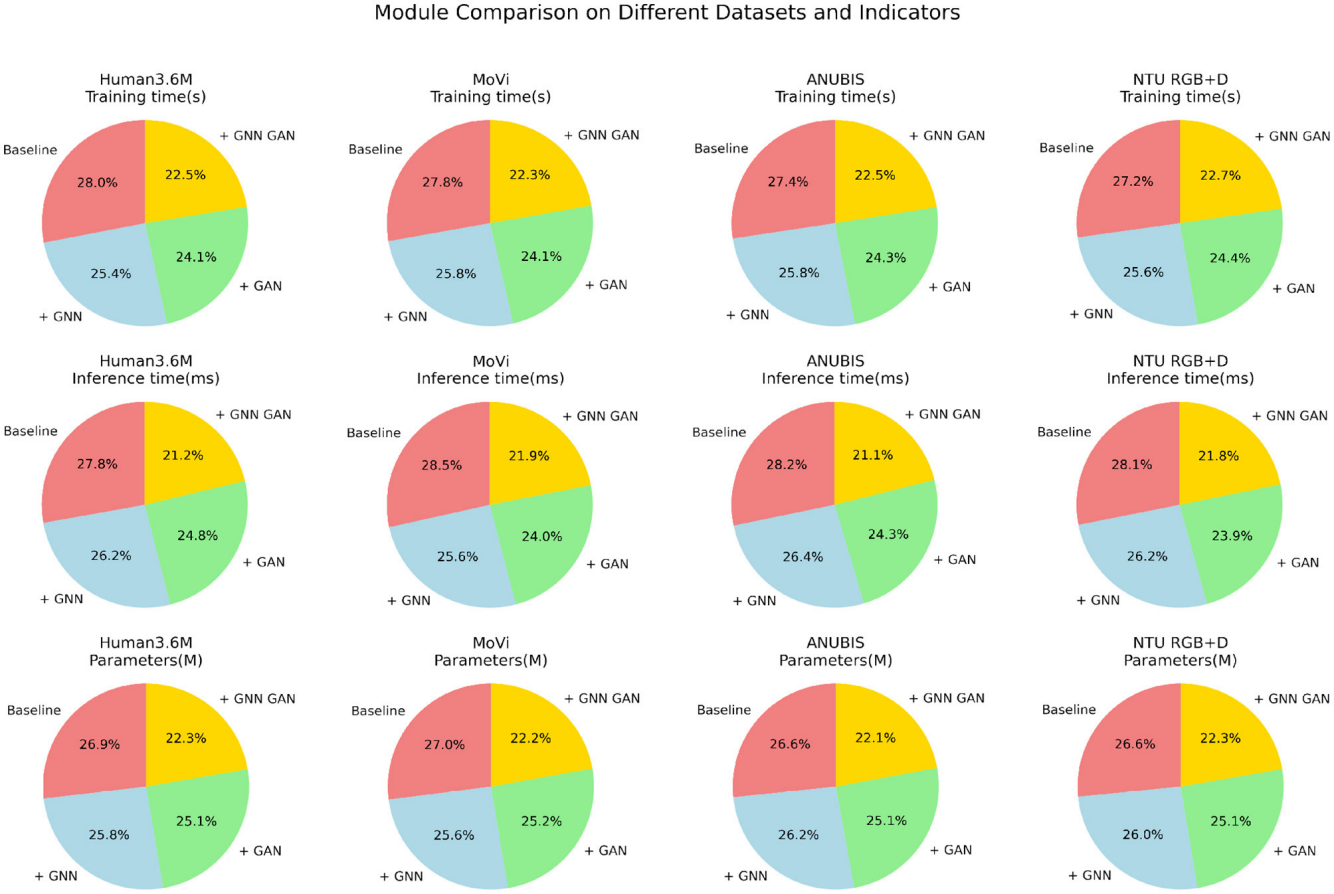
Comparative visualization of training time, inference time, and parameters indicators under different modules based on four data sets.

The data from Tables 7, 8 illustrates that with the continuous optimization of the model structure, various operational efficiency indicators are consistently improving. The simple baseline structure performs poorly in terms of training, inference time, and parameter quantity. After incorporating the GNN module, all indicators experience certain improvements, such as a reduction in training time by over 5 seconds and a decrease of around 10 million parameters. Similarly, the independent use of GAN also reduces these indicators, with an inference time improvement of up to 20 ms. However, the joint learning framework of GNN and GAN that we adopted significantly enhances efficiency. Under this framework, training and inference times are the lowest for each dataset, with a reduction of over 10 s in training time compared to the baseline and a faster inference by nearly 30 ms. Additionally, the parameter count is minimized, requiring only 80% of the baseline module's quantity. This strongly indicates that the novel approach proposed in our work makes the model learning more efficient, significantly saving computational resources while ensuring accuracy. Overall, as the model structure continues to be optimized, from baseline to the introduction of various modules, and further to our proposed joint framework, operational performance consistently improves. This suggests that our method will have advantages over previous approaches in practical applications. Finally, we have visualized the data results from Tables 7, 8 in Figure 9.

**Table 7:** Comparison of training time, inference time, and parameters indicators under different modules based on Human3.6M and MoVi data sets.

**Model**	**Datasets**					
		**Human3.6M Dataset (Ionescu et al.**, **2013****)**			**MoVi Dataset (Ghorbani et al.**, **2020****)**		
		**Training time (s)**	**Inference time (ms)**	**Parameters (M)**	**Training time (s)**	**Inference time (ms)**	**Parameters (M)**
Baseline	53.51	142.75	266.18	54.29	143.24	264.99
+ gnn	48.48	134.34	255.71	50.43	128.68	251.47
+ gan	46.04	127.22	248.44	47.06	120.65	246.96
+gnn gan	42.89	109.07	220.97	43.48	110.21	217.69

**Table 8:** Comparison of training time, inference time, and parameters indicators under different modules based on ANUBIS and NTU RGB+D data sets.

**Model**	**Datasets**					
		**ANUBIS Dataset (Qin et al.**, **2022****)**			**NTU RGB+D Dataset (Shahroudy et al.**, **2016****)**		
		**Training time (s)**	**Inference time (ms)**	**Parameters (M)**	**Training time (s)**	**Inference time (ms)**	**Parameters (M)**
Baseline	50.17	135.27	248.97	51.33	138.88	251.08
+ gnn	47.22	126.37	244.81	48.34	129.38	246.19
+ gan	44.60	116.34	234.34	46.05	118.02	237.39
+gnn gan	41.27	101.08	206.38	42.90	107.67	210.47

Overall, through this series of experiments, we have conducted a comprehensive and detailed comparative analysis of the performance of different human action recognition models on four representative datasets. Across various dimensions, including evaluation metrics, operational efficiency, and diverse model designs, the advantages of our proposed method have been thoroughly validated. In comparison to previous research approaches, we leveraged the synergistic benefits of GNN and GAN technologies to devise a novel joint learning framework. Experimental results indicate that this framework can effectively harness the strengths of these two technologies, significantly optimizing model efficiency while ensuring recognition accuracy. Furthermore, with continuous improvements in the modules, progressing from individual applications to joint usage, both human action recognition capabilities and operational efficiency have seen noticeable enhancements. This strongly demonstrates the importance and innovative value of the new approach proposed in our work within this field. By delving into an in-depth analysis of extensive experimental data, this paper provides a systematic demonstration of the impact of module optimizations and overall framework design on model performance. This serves as a valuable reference for subsequent work aiming to bring innovative designs to this task.

## 5 Discussion and conclusion

The conclusion and discussion section marks the exciting conclusion of our research, providing a profound summary of the entire paper. Throughout the discourse, we focused on the key technologies of skeleton motion analysis and the performance of our proposed method based on Transformer and Graph Neural Networks (GNN) in optimizing sports training and preventing injuries. This chapter delves into a comprehensive discussion of the research problem, the methods employed, and the experimental results, aiming to showcase the contributions and achievements of our study.

With the increasing demand for health-related physical activities in modern society, skeleton motion analysis has become crucial in enhancing exercise effectiveness and reducing potential injuries. Our research integrates advanced Transformer models, Graph Neural Networks (GNN), and Generative Adversarial Networks (GAN) technologies to optimize sports training and improve injury prevention. Building upon a deep understanding of this field, we conducted a series of experiments and provided a thorough analysis of the results, revealing the superiority and potential application value of the proposed method compared to traditional approaches.

Our study focuses on skeleton motion analysis, employing advanced Transformer models, Graph Neural Networks, and Generative Adversarial Networks to comprehensively enhance sports training and injury prevention. By integrating global contextual information, local motion features, and generating more realistic and diverse motion sequences, our method achieved significant improvements across multiple key indicators. This research not only demonstrates technological innovation but also provides new technical support for the field of neuromusculoskeletal modeling.

The innovation of this study lies in the sophisticated integration of Transformer, Graph Neural Networks, and Generative Adversarial Networks, enabling our method to comprehensively and accurately capture the features of skeleton motions. On a theoretical level, we experimentally demonstrated the significant effects of the new method in optimizing sports training and preventing injuries, offering new perspectives and methods for research in related fields. In practical applications, our proposed algorithm provides finer and more personalized technical support for the prevention of injuries and sports training in the neuromusculoskeletal system, driving advancements in this field. This research showcases technological foresight, providing new perspectives and enriching research content for the progress of neuromusculoskeletal models.

In experiments, we utilized multiple datasets, including the “Human3.6M Dataset,” “MoVi Dataset,” “ANUBIS Dataset,” and “NTU RGB+D Dataset,” to comprehensively validate the performance of the proposed method. This study aims to provide new ideas and technical support for the development of skeleton motion analysis. By comparing with traditional methods, our approach achieved significant improvements in various metrics such as specificity, accuracy, recall, and *F*1-score. The experimental results demonstrate the superiority of our method in skeleton motion analysis tasks compared to traditional approaches. Specifically, our method showed significant improvements in specificity, accuracy, recall, and F1-score, increasing by ~6%, around 5%, around 6%, and reaching above 89%, respectively. These results fully validate the effectiveness and superiority of our method in optimizing sports training and preventing injuries. Moreover, our method exhibits significant advantages in capturing global contextual information, modeling local motion features, and generating diverse motion sequences. This provides a solid theoretical and experimental foundation for improving the precision of sports training and the effectiveness of injury prevention.

Despite the satisfactory achievements of this study, there are inevitably some limitations. Firstly, our method may face challenges in handling certain complex scenarios, necessitating further consideration of diversity and complexity issues. Secondly, the scale and diversity of experimental datasets may affect the generalization ability of the model. These limitations provide directions for future research. Finally, this study did not consider noise and interference in actual motion scenarios, and the adaptability to real-world applications needs further verification.

Based on existing research and experimental results, future work can be expanded in the following areas: Firstly, further optimization of the model structure and parameters can be conducted to enhance the model's performance on specific skeleton motions. Secondly, expanding the experimental dataset will better validate the model's generalization ability. Finally, an in-depth exploration of the application of Generative Adversarial Networks in skeleton motion analysis can be pursued to further improve the model's robustness and adaptability.

In summary, this study proposes an innovative and effective skeleton motion analysis method through the intricate integration of Transformer, Graph Neural Networks, and Generative Adversarial Networks. Significant experimental results have been achieved in the field of neuromusculoskeletal models. In-depth comparisons and analyses demonstrate the apparent superiority of our method in optimizing sports training and preventing injuries. However, the research still faces challenges and limitations, providing directions for future in-depth investigations. Overall, this study injects new ideas and methods into the direction of injury prevention and sports training in neuromusculoskeletal models, offering valuable insights for future research and applications. We look forward to active participation from more scholars and practitioners in the in-depth exploration of this field, collectively advancing the development of skeleton motion analysis technology. By providing intelligent and effective fitness guidance and sports rehabilitation support, we believe advancements in this field will bring a healthier and more scientific sports experience to sports enthusiasts. We are confident in the future development of the neuromusculoskeletal models field and believe this research will provide beneficial guidance and inspiration for related research and applications.

## Data availability statement

The original contributions presented in the study are included in the article/supplementary material, further inquiries can be directed to the corresponding author.

## Author contributions

JZ: Data curation, Funding acquisition, Investigation, Project administration, Writing – review & editing. ZY: Conceptualization, Data curation, Formal analysis, Resources, Writing – review & editing. MR: Investigation, Methodology, Project administration, Resources, Writing – review & editing. GM: Data curation, Investigation, Methodology, Project administration, Resources, Writing – original draft, Writing – review & editing.
